# Root morphology and exudate availability are shaped by particle size and chemistry in *Brachypodium distachyon*

**DOI:** 10.1002/pld3.207

**Published:** 2020-07-02

**Authors:** Joelle Sasse, Suzanne M. Kosina, Markus de Raad, Jacob S. Jordan, Katherine Whiting, Kateryna Zhalnina, Trent R. Northen

**Affiliations:** ^1^ Environmental Genomics and Systems Biology Lawrence Berkeley National Laboratory Berkeley CA USA; ^2^ Joint Genome Institute Lawrence Berkeley National Laboratory Berkeley CA USA; ^3^ Department of Plant and Microbial Biology University of Zurich Zurich Switzerland

**Keywords:** *Brachypodium distachyon*, particle chemistry, particle size, *Pseudomonas fluorescens*, rhizosphere, root exudation, root morphology

## Abstract

Root morphology and exudation define a plants’ sphere of influence in soils. In turn, soil characteristics influence plant growth, morphology, root microbiome, and rhizosphere chemistry. Collectively, all these parameters have significant implications on the major biogeochemical cycles, crop yield, and ecosystem health. However, how plants are shaped by the physiochemistry of soil particles is still not well understood. We explored how particle size and chemistry of growth substrates affect root morphology and exudation of a model grass. We grew *Brachypodium distachyon* in glass beads with various sizes (0.5, 1, 2, 3 mm), as well as in sand (0.005, 0.25, 4 mm) and in clay (4 mm) particles and in particle‐free hydroponic medium. Plant morphology, root weight, and shoot weight were measured. We found that particle size significantly influenced root fresh weight and root length, whereas root number and shoot weight remained constant. Next, plant exudation profiles were analyzed with mass spectrometry imaging and liquid chromatography–mass spectrometry. Mass spectrometry imaging suggested that both, root length and number shape root exudation. Exudate profiles were comparable for plants growing in glass beads or sand with various particles sizes, but distinct for plants growing in clay for in situ exudate collection. Clay particles were found to sorb 20% of compounds exuded by clay‐grown plants, and 70% of compounds from a defined exudate medium. The sorbed compounds belonged to a range of chemical classes, among them nucleosides, organic acids, sugars, and amino acids. Some of the sorbed compounds could be desorbed by a rhizobacterium (*Pseudomonas fluorescens* WCS415), supporting its growth. This study demonstrates the effect of different characteristics of particles on root morphology, plant exudation and availability of nutrients to microorganisms. These findings further support the critical importance of the physiochemical properties of soils when investigating plant morphology, plant chemistry, and plant–microbe interactions.

## 1 INTRODUCTION

Plant roots shape their environment in various ways and are in turn shaped by physiochemical properties of the surrounding soil. Roots affect soil by dislocating particles, by polymer production, and by the release of a wide variety of small molecules (root exudation). Root exudates alter pH and the chemical composition around roots. Overall, these processes result in the formation of larger soil aggregates which increase water‐holding capacity (Six, Bossuyt, Degryze, & Denef, 2004) and influence the stability of soil organic carbon (Sokol, Sanderman, & Bradford, 2018). Plant‐induced changes in chemistry can lead to weathering of minerals (Uroz, Kelly, Turpault, Lepleux, & Frey‐Klett, 2015) and alter the composition of microbial communities (Carson, Rooney, Gleeson, & Clipson, 2007). Further, root exudates can enhance root penetration of soils (Oleghe et al., 2017). Although the effects of plant presence on soils and microbial communities have been a major research questions for over a century (Brink, 2016; Sokol et al., 2018; Haichar 2018; Vives‐Peris, Ollas, Gómez‐Cadenas, & Pérez‐Clemente, 2019), relatively few studies have sought to understand the physiochemical effects of plant growth substrates on plant physiology and exudation.

Plant morphology and exudations can be influenced by both physical and chemical properties of soil particles. Typically, particles range from small (<50 µm) to large (>2 mm) and determine physical parameters such as water‐holding capacity of soils (Rellán‐Álvarez, Lobet, & Dinneny, 2016; Six et al., 2004; Six & Paustian, 2014). It has been shown that 1‐mm beads reduce root and shoot growth, elevate root:shoot ratios, and alter root morphology of maize when compared to hydroponic growth (Boeuf‐Tremblay, Plantureux, & Guckert, 1995; Groleau‐Renaud, Plantureux, & Guckert, 1998; Veen, 1982). Root morphology can be altered by adsorption of root exudate metabolites to substrate such as activated carbon (Caffaro, Vivanco, Gutierrez Boem, & Rubio, 2011). Natural environments, such as soils, can also affect both root exudate profiles and morphology (Neumann et al., 2014); however, determination of causal factors may be confounded by variables such as granule size, chemistry, and microbial community composition. Soil minerals differ in structure (e.g., accessible surface areas) and surface charge, thereby governing their interaction with dissolved organic compounds (Swenson, Bowen, Nico, & Northen, 2015a). Results suggest that substrate chemistry can alter exudation. For example, aluminum ions present in stone wool are thought to increase exudation of organic acids in maize (Kamilova et al., 2006).

In contrast to particle chemistry, the effect of particle size on exudation is less clear. Particle size could alter exudation in multiple ways. (a) Particle size reduces root growth rates, which limits exudate dispersal. In addition, diffusion depends on substrate size and can thus be a limiting factor for dispersal of exuded compounds (Kim, Silk, & Cheer, 1999). (b) Exudates are mainly produced by root tips (Holz, Zarebanadkouki, Kaestner, Kuzyakov, & Carminati, 2018): when root morphology is altered by particle size, the number and structure of root tips can be altered, possibly changing the quality and quantity of exudates. (c) Particle size also influences the composition of microbial communities (Certini, Campbell, & Edwards, 2004). Microbes differentially metabolize exudates (Zhalnina et al., 2018) and secrete secondary metabolites (Etalo, Jeon, & Raaijmakers, 2018), further changing the presence of exuded compounds. d) Particle size can modulate plant chemistry (Etalo et al., 2018).

Here, we investigate the effect of particle size and chemistry on root morphology and exudation in *Brachypodium distachyon*. Specifically, we asked three questions: a) whether root morphology of a model grass is altered in physically restricted conditions as observed in other species, b) if and how the exudate profile changes with particle size, and c) how root morphology and exudation are influenced by substrate chemistry. To facilitate these studies, we used a sterile environment enabling us to focus on plant metabolism without the additional layer of microbial metabolism present in a natural environment. *B. distachyon* growth and exudation profiles were comparted on various inert substrates in a range of defined particle sizes (sand, glass beads, and clay) relative to hydroponic solution. We found that particle size had a significant effect on root weight and root length; however, particle size did not influence composition of root exudates. A defined mixture of soil metabolites was used to evaluate sorption to particles. We further demonstrated that clay sorbed a large degree of exudates, altering the amount of exudates freely available around root. These clay‐sorbed exudates could further support growth of a rhizobacterium. Our results highlight the importance of considering soil structure and chemistry when studying plant–soil interactions.

## 2 RESULTS

### 2.1 Metabolite sorption to substrates

Different particle sizes and surface chemistries were chosen to investigate how root morphology and exudation is affected in various plant growth substrates. The particle sizes chosen corresponded to large, intermediary, and small particles (Six, Paustian, Elliott, & C C, 2000) (see Materials and Methods). Glass beads were chosen as an inert system with defined sphere diameter, sand as an inert and natural system, and clay as a natural system with a reactive surface. The mineral composition of the sand substrates was determined as more than 98% quartz, whereas the clay was a mixture of 51% opal‐CT, 37% mica‐illite, 10% quartz, and trace amounts of K‐feldspar and calcite (Figure S1).

**FIGURE 1:**
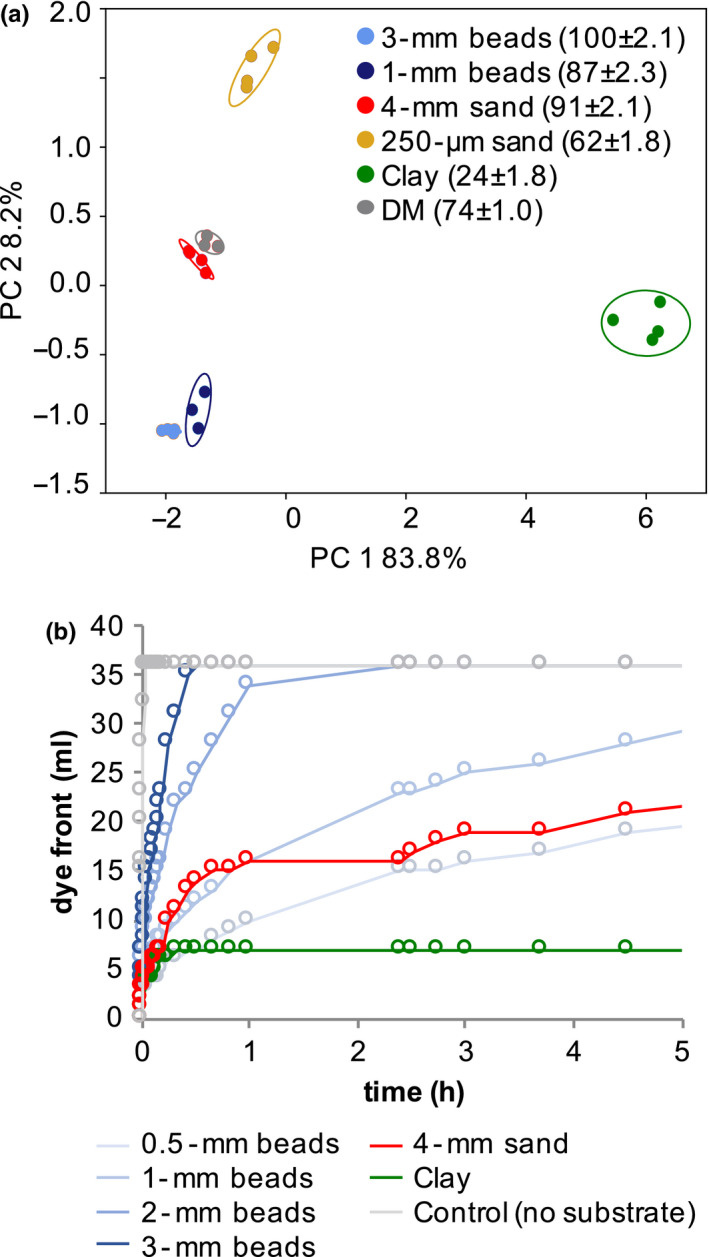
Physiochemical substrate properties (a) Principal component (PC) analysis of defined medium (DM) metabolites which were incubated with different substrates. The metabolite relative abundance in percent was normalized by defined medium incubation without substrate, and the recovery rate is given in brackets (means ± *SEM*, *n* = 3). (b) Diffusion of colorimetric dye through various substrates as determined by the advancement of the dye front over time

The chemical properties of the substrates were assessed by determining the sorption of a mixture (termed “defined medium” herein) of more than forty metabolites belonging to various chemical classes that have also been found in root exudates (amino acids, organic acids, sugars, nucleobases, nucleosides, and others, see Table S2). The recovery rate of the various metabolites from the glass beads and the 4‐mm sand was comparable to the defined medium control without substrate, whereas the recovery rate from the 250‐µm sand and from clay were lower by approximately 30% and 70%, respectively (Table S2, Figure S6). Consistently, differences between clay and other substrates explained 84% of the variance in a principal component analysis, and only 8% of the variance accounts for differences between the control, glass beads, and sand (Figure 1a). The metabolites depleted by clay belonged to a variety of chemical classes, among them charged compounds, such as organic acids and ammonium salts (carnitine, acetylcholine), and other nitrogenous compounds (amino acids, nucleosides), and of comparatively polar compounds such as sugars (Table S2). We confirm that as expected, clay particles sorb a variety of metabolites from the defined medium.

### 2.2 Substrate diffusion rates

Exudation could be limited by diffusion in our experimental systems with small particle sizes. A diffusion test with a dye resulted in fastest diffusion in controls without substrate added (Figure 1b). The diffusion rate of the dye decreased with lower diameters in glass beads and followed a logarithmic trend. In 4‐mm sand, the diffusion rate was initially similar to 1‐mm glass beads, but then resembled more 0.5‐mm beads. For clay, diffusion similar to 1‐mm or 0.5‐mm beads was observed initially, but subsequently, the dye front ceased moving, likely due to sorption of the negatively charged dye.

Based on this analysis, in our experimental setup, exudates would require a minimum diffusion rate of 1.25 cm/h to reach the edge of the glass jar in which the plants were grown (10‐cm jar diameter, 2‐hr exudate collection, plants positioned equidistant between center and edge). Thus, diffusion was not limiting in glass beads with a diameter equal to or >1 mm, but might be limited in substrates with smaller diameters (Table S1). This confirms, that as expected, sand and glass beads are inert substrates, whereas clay strongly sorbs a variety of metabolites. In addition, exudation may be limited by diffusion in substrates with particle sizes smaller than 1 mm.

### 2.3 Root morphological changes in substrates

**FIGURE 2:**
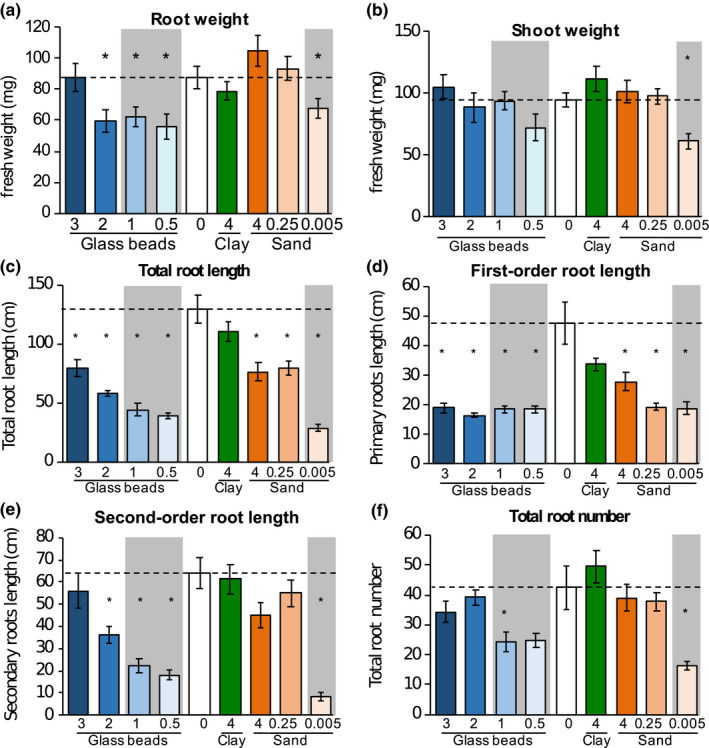
Tissue weight and root morphology Root (a) and shoot fresh weight (b) of 3‐week‐old *B. distachyon* growing in different substrates. Root morphology was assessed as total root length (c), which is the sum of first‐order root length (d), second‐order root length (e), and third‐order root length (Figure S2); and the total root number (f), which is the sum of first, second, and third‐order root numbers (Figure S2). Particle sizes are indicated in cm. Data are means ± *SEM*, *n* > 5. Significant differences are displayed as asterisks (*, ANOVA, *p* = .05) of substrates compared to hydroponic control (0, dashed line). Gray areas: plants with weight and root morphology distinct from hydroponic controls

The aforementioned substrates were used to investigate how *Brachypodium distachyon* root morphology and exudation was affected in these experimental systems compared to a hydroponic control. Plants were grouped according to their behavior in the different substrates: plants with weights and root morphology similar to hydroponic controls were termed “big” (big beads: 3 mm, 2 mm; big sand: 4 mm, 250 µm; clay), and plants with distinct weight and root morphology were termed “small” (small beads: 1 mm, 0.5 mm, small sand: 5 µm. Figure 2, gray areas).

The root fresh weight of plants grown in 3‐mm glass beads, 4‐mm sand, 250‐µm sand, and clay was comparable to the hydroponic control, whereas roots grown in 2‐mm, 1‐mm, and 0.5‐mm glass beads and 5‐µm sand were significantly smaller (ANOVA/Tukey test, alpha = 0.05, Figure 2). The shoot fresh weight of plants grown in 5‐µm sand were significantly smaller compared with plants grown in hydroponics, and all other conditions (Figure 2). The altered root and shoot weights resulted in decreased root/shoot ratios for clay, and 2‐mm and 1‐mm glass beads‐grown plants, and an increased ratio of 5‐µm sand‐grown plants (Figure S2).

Root length and number were assessed for first‐order roots (primary and crown roots), second‐order roots (laterals of primary and crown roots), and higher order roots. The total root length correlated with particle size, with maximal length for hydroponically and clay‐grown roots, approximately 30% shorter root systems for 3‐mm beads‐, 4‐mm sand‐ and 250‐µm sand‐grown roots, and 50% or shorter root systems for 1‐mm beads‐, 2‐mm beads‐, 0.5‐mm beads‐, and 5‐µm sand‐grown roots (Figure 2). First‐order root lengths were significantly decreased by more than half for all substrates except for clay and 4‐mm sand (Figure 2), whereas the second‐order root length was decreased by 40%–70% in 2‐mm beads‐, 1‐mm beads‐, and 0.5‐mm bead‐grown roots, and by ~85% in 5 µm sand‐grown roots (Figure 2). Higher order root lengths varied more within one experimental treatment, with a trend for higher total lengths in hydroponics and clay compared with glass beads and sand, and significantly lower lengths in 5‐µm sand (Figure S2).

Interestingly, root length had a higher Pearson correlation coefficient when correlated with particle size than root numbers. Only roots grown in 1‐mm beads and 5‐µm sand showed a statistically significant reduction in root number compared with hydroponic controls, which is a result of the large variability in total root number of hydroponically grown plants (21 to 84 roots, Figure 2, Figure S3). The observed reduction in root number originated from a reduced number of secondary and higher order roots (Figure 2, Figure S2).

A correlation analysis between root and shoot weight, total root length, and total root number of all samples showed a significant correlation of all parameters investigated (Pearson correlation coefficients, *p* = .01, Figure S4). Root weight and length, and to a lesser degree root number, correlated with particle size.

Overall, clay‐grown plants were most similar to hydroponically grown plants regarding tissue weight and root morphology. Plants grown in 3‐mm glass beads or 4‐mm sand had comparable fresh weight compared to the aforementioned plants, but slightly reduced total root length driven by a reduction in first‐order root length. Plants grown in 1‐mm and 0.5‐mm glass beads exhibited reduced root weight and root length, caused by a reduction in first and second‐order root length. Plants grown in 5‐µm sand exhibited the largest reduction in tissue weight, root length, and number.

### 2.4 Spatially distinct exudation patterns

**FIGURE 3:**
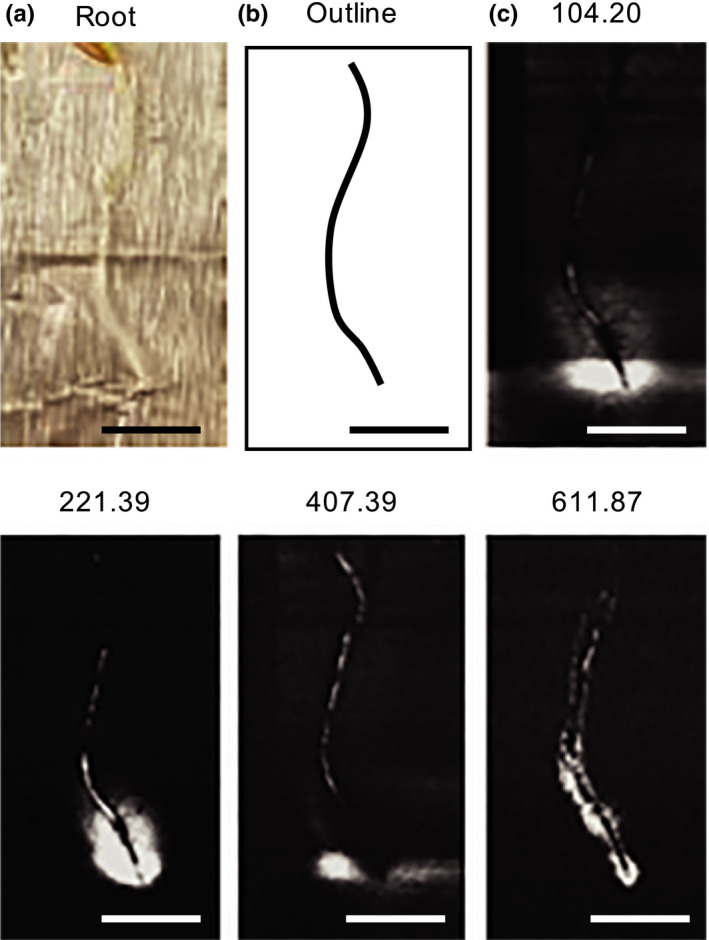
Spatial exudation patterns Plants were incubated for 6 hr to allow for exudation (a). A traced outline of the root is displayed (b). Root‐associated ion patterns (50–2000 Da) of several ions were observed with Mass Spectrometry Imaging. The patterns of several ions are displayed here, with their m/z indicated above the panels (c). The panels are a subset of Figure S5. Scale bars: 1 cm

To investigate whether changes in root morphology might affect exudation profiles, spatial patterns of exudation were investigated using matrix‐assisted laser desorption/ionization mass spectrometry (MALDI). A total of 24 ions were detected in the vicinity of roots (Figure 3, Figure S5). It was not possible to confidently identify these ions given that the MALDI used is not suited for fragmentation of low m/z ions and the fact that MALDI often results in different ions versus the electrospray ionization used in our liquid chromatography–mass spectrometry analyses. However, despite this lack of identifications, our results suggest differences in spatial patterns of chemical components. Some ions showed higher abundances around root tip and elongation zone, supporting a role of these young root tissues in exudation. Other ions were detected along most of the root axis, suggesting exudation also from older root parts, whereas the location of other ions overlapped with the location of the root, which could either indicate short diffusion distances, or association with the cell wall. Overall, these data suggest that multiple tissues are involved in exudation.

### 2.5 In situ versus in vitro exudate profiles

To investigate whether altered root morphology and various spatial exudation patterns altered overall exudation profiles, exudates were collected in situ (plants growing in substrates) and in vitro (plants removed from substrates prior to exudate collection). The first collection approach (in situ) generated exudation profiles shaped by plant metabolism and particle chemistry, whereas the second collection approach (in vitro) generated exudate profiles shaped only by plant metabolism. With both collection steps, approximately 100 metabolites were identified based on comparison of retention times, exact mass, and MS/MS fragmentation patterns compared with authentic standards. The metabolites included organic acids, amino acids and derivatives, sugars and other carbohydrates, nucleic bases, nucleosides, and derivatives. Multivariate statistical analysis revealed that 46% of in situ exuded metabolites were significantly different in pairwise comparisons, compared with 30% of in vitro exuded metabolites (ANOVA/Tukey test, *p* = .05, Figure S6). A similar result is evident in hierarchical clustering analysis, with in situ‐collected exudates clustering according to biological replicates, and in vitro‐collected exudates clustering in less distinct patterns (Figure S7b).

**FIGURE 4:**
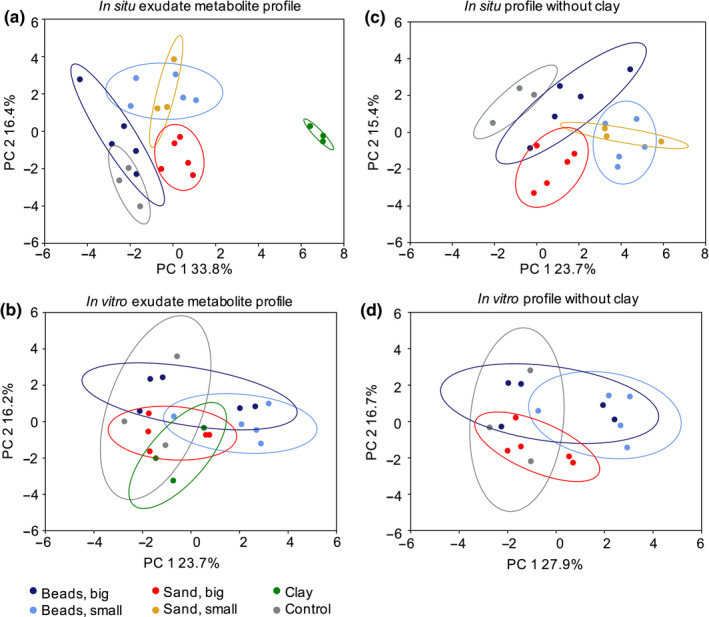
In situ and in vitro exudate metabolite profiles Principal component (PC) analysis of root exudate metabolite profiles collected in situ (a) or in vitro (b). Substrates are grouped as follows: beads big: 3‐mm and 2‐mm glass beads; beads small: 1‐mm and 0.5‐mm glass beads; sand big: 4‐mm and 250‐µm sand; sand small: 5‐µm sand. The small sand dataset is missing from the in vitro analysis due to a technical error. Control: exudates from hydroponics. PC analyses of single substrates and hierarchical clustering of the data are displayed in Figure S7

Most of the variation for in situ exudates was explained by differences between exudates collected in clay, compared with other conditions, which is evident from a principal component analysis (Figure 4). Similarly, in pairwise comparisons, clay‐collected exudates showed the most distinct metabolites (3%–24%), followed by 250 µm sand‐collected exudates with 5%–18% distinct metabolites (Figure S6). In contrast to in situ exudates, in vitro exudates exhibited similar metabolite profiles when analyzed with a principal component analysis (Figure 4, Figure S7), and fewer metabolites had statistically significant abundances in pairwise comparisons (Figure S6). Notably, in vitro exudates of clay‐grown plants showed a comparable number of distinct metabolites in pairwise comparisons with plants grown in other substrates, suggesting that the in situ differences observed between exudate profiles of clay‐grown plants and plants grown in other conditions resulted from the presence of the clay, and not from an altered plant metabolism.

### 2.6 Analysis of the in situ effects of large versus small particles on exudate profiles

To further investigate differences in in situ‐collected exudates, exudate profiles of the groups by their particle sizes and “big beads,” “small beads,” “big sand,” and “small sand” were compared (grouping according to root morphology phenotypes) (Figure 4, plots for individual substrates are shown Figure S7). In a principal component analysis, exudate profiles of hydroponic and “big beads” exudates overlapped, whereas “big beads” versus “small beads” and “big sand” versus “small sand” separated (Figure 4). Pairwise comparisons showed thirteen distinct metabolites between “big beads” and “small beads,” and three distinct metabolites between “big sand” and “small sand.” Two thirds of metabolites were more abundant in “big beads” than “small beads,” among them nucleobases and derivatives, as well as organic acids. Four phenolic acids were more abundant in “small beads” versus “big beads.” In “small sand,” two nitrogenous compounds were higher abundance than in “big sand,” a nucleobase, (cytosine), and an amino acid derivative (carnitine) (Figure S8).

### 2.7 In situ charged versus uncharged substrates exudate profiles

**FIGURE 5:**
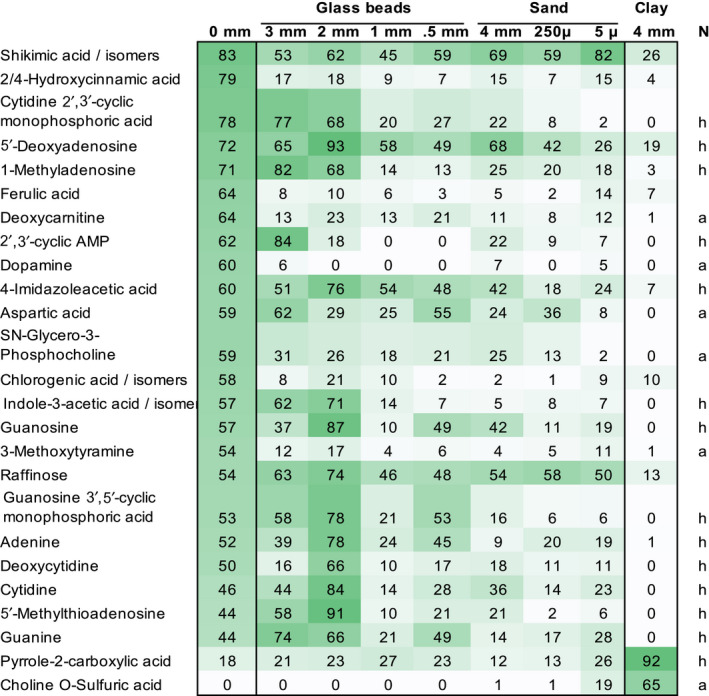
Distinct metabolites of in situ clay Heatmap of metabolite abundances significantly different between exudates collected in situ from clay‐grown and hydroponically grown plants (black boxes). All substrates are displayed for comparison. Values indicate averaged peak heights, scaled to the maximum intensity for each compound. Metabolites are sorted in descending abundance for hydroponic exudates (0 mm). Data are means of 3 biological replicates, ANOVA, *p* = .05. Nitrogenous compounds are labeled as containing heterocyclic (h) or aliphatic (a) nitrogen. The full dataset is given in Table S1

To further examine the differences between clay‐grown and hydroponically grown in situ exudates, a multi‐variant test was used to compare metabolite abundances between the two conditions. Ninety‐two percent (23 of 25 metabolites) were significantly less abundant in clay than in hydroponics (Figure 5). Most of these metabolites were nitrogenous, with more than half containing a heterocyclic nitrogen group. Among these metabolites were nucleic bases, nucleosides and derivatives with acidic groups, amino acids with acidic and/or basic groups, and linear as well as phenolic organic acids. Two nitrogenous metabolites, an organic acid and choline‐O‐sulfate with an acidic and a basic group, were more abundant in clay‐collected exudates (Figure 5). These compounds were not detected in exudates of hydroponically grown plants, in in vitro‐collected exudates of clay‐grown plants, or in clay control samples without plants (Table S2), which suggests that these compounds were released from clay only in the presence of plants.

### 2.8 A plant‐beneficial microbe can grow on clay‐sorbed metabolites

Since clay particles were found to strongly sorb exudate metabolites, we wondered whether the sorbed metabolites were accessible to a plant‐associated bacterium, supporting microbial growth. Thus, we first determined the desorption rate of metabolites from the various substrates by determining the metabolite recovery rate from glass beads, sand, and clay incubated with defined medium. As shown previously (Figure 1), the metabolite recovery rate was comparable between the no substrate control and glass beads (63%–80% recovery), lower in sand (31%–51% recovery), and the lowest for clay (27%, Figure S9). The metabolite recovery from washes was 4%–14% for all substrates, indicating all substrates had similar desorption rates.

**FIGURE 6:**
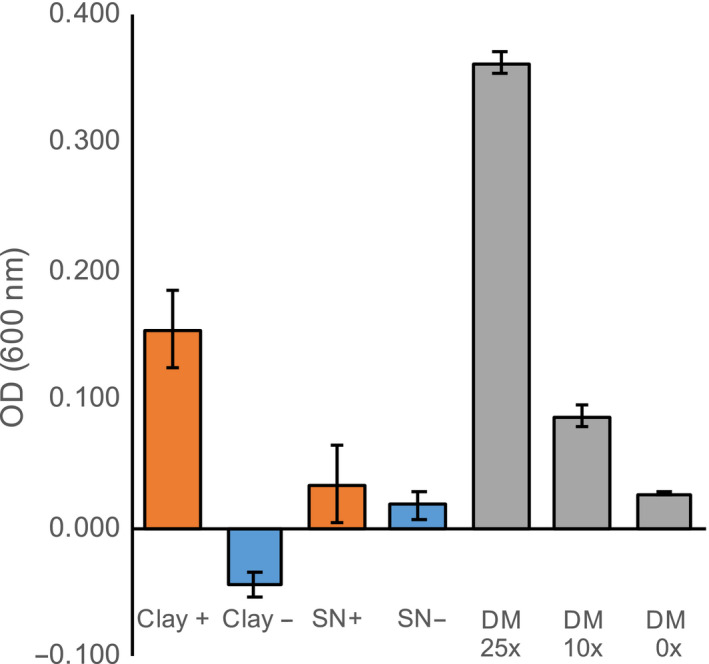
Rhizobacterium grows on clay‐adsorbed metabolites Optical density (OD at 600 nm) of *P. fluorescens* grown on clay pre‐incubated with 50× defined medium (DM, Clay +) or 0× DM (Clay −), or in supernatant of clay pre‐incubated with 50× DM (SN +) or 0× DM (SN −). All ODs are means ± *SEM* (*n* = 3), normalized by OD of clay without bacteria (see Figure S10 for entire dataset). *P. fluorescens* growth in different concentrations of DM without substrate is given as comparison

Growth of the rhizobacterium *Pseudomonas fluorescens* on sand or glass beads pre‐incubated with defined medium resulted in the same optical density (OD) change as growth on particles pre‐incubated with water (Figure S10), indicating that these substrates did not retain metabolites supporting growth. Incubation of the bacterium with clay pre‐incubated with defined medium however did result in bacterial growth. As the control incubation of clay with defined medium (without bacteria) also showed a small increase in OD, presumably as a result of fine particles, the data presented in Figure 6 are normalized by no‐bacterial clay control samples (Figure S10 presents the raw data). These data show an increase in OD of bacteria grown on clay pre‐incubated with defined medium, indicating that the bacteria are able to utilize the sorbed metabolites for growth.

As an additional control experiment, the pre‐incubated clay was incubated with water for three days under sterile conditions (the same timing as for the bacterial growth experiment), allowing for desorption of metabolites from clay particles. The supernatant was subsequently pipetted into a new well, and bacteria were added and allowed to grow for another three days. This experiment resulted in no‐bacterial growth (Figure 6, Figure S10), suggesting that bacterial presence is needed to desorb metabolites from clay particles. We conclude that this particular rhizobacterium is capable of desorbing exudate metabolites from clay to support growth.

## 3 DISCUSSION

### 3.1 Root weight and morphology are influenced by substrate size

Growth of *B. distachyon* in particles with different sizes resulted in various morphological changes. A decrease in particle size resulted in decreased root weight, total root length, and in total root number, although the last parameter correlated less strongly (Figure 2, Figure S4). Root weight correlated positively with shoot weight, total root length, and total root number, indicating a dependency of the different parameters.

Notably, the morphology of *B. distachyon* grown in glass beads or sand was not directly comparable: Plants grown in 5‐µm sand had higher root weight and total root length than plants grown in 0.5‐mm glass beads. The three‐dimensional particle arrangement and other differences between the substrates, such as texture, might account for root morphological differences observed between glass bead‐ and sand‐grown plants.

For glass bead‐grown plants, the reduction in total root length was caused by a reduction in second‐order root length, whereas the primary order root length was reduced in all sizes smaller than 3 mm. These trends for reduced root weight and root length but not root number are in line with observations made for maize grown in 1 mm versus hydroponic conditions (Boeuf‐Tremblay et al., 1995; Groleau‐Renaud et al., 1998; Veen, 1982). However, these previous studies noted an even larger decrease in shoot than in root weight, whereas *B. distachyon* shoot weight did not change significantly in our experimental conditions. Similar results were found for lettuce grown in three different soils, where root fresh weight and morphology changed, but shoot weight was not affected (Neumann et al., 2014). The constant *B. distachyon* shoot weight might indicate sufficient nutrient uptake even by smaller root systems in the environments investigated. Thus, in future studies, it might be interesting to evaluate how the different root systems as generated here with different substrate sizes further respond to altered nutrient levels.

One could expect an additional change in root morphology with different nutrient starvation conditions, for example, root systems optimized for phosphate scavenging form numerous, short lateral roots, whereas roots optimized for nitrogen uptake exhibit fewer, but long lateral roots (Rellán‐Álvarez et al., 2016). Phosphate movement is hindered by particles with a charged substrate, whereas nitrate movement is less affected by soil chemistry (Rellán‐Álvarez et al., 2016). Thus, changes in root morphology of plants grown in phosphate‐limited clay might be distinct from plants grown in phosphate‐limited sand or glass beads. Plants grown in soil may exhibit additional changes in root morphology and metabolism, as shown for *B. distachyon* grown in a sterile soil extract, which showed reduced root length, and elongated root hairs and which depleted a variety of metabolites from soil extract (Sasse, Kant, Cole, Klein, & New, 2018). Root systems further respond to local alterations in soil structure, such as to the presence of micro‐ or macropores, or to air pockets (Rellán‐Álvarez et al., 2016). Investigations of local root morphology responses in heterogeneous settings with multiple, defined substrate sizes and chemistries will thus shed more light onto how plants respond to soil physiochemistry on a spatial and time scale. Multiple systems exist in which such experiments could be attempted, ranging from EcoFAB model systems (Gao et al., 2018) to rhizotron designs (Grossmann et al., 2011; Parashar & Pandey, 2011; Rellán‐Álvarez et al., 2015).

### 3.2 Exudation across the root system

*B. distachyon* exhibited significantly altered root morphology when grown in particles with various sizes, with root weight, and root lengths differing between conditions. The exudate profile however was very similar for these plants when collected in vitro (Figure 4), and exudate extraction volumes were normalized by root fresh weight before measurement. Thus indicating that exudation per root fresh weight is constant. As root weight correlated with both, total root length and with total root number, an additional method was needed to determine whether the number of roots or the root length was important for exudation.

In the literature, root tips are often mentioned as predominant sites of exudation for several reasons: a) cell wall suberization of this young tissue is still low (Jones, Nguyen, & Finlay, 2009), b) exudates have been imaged around root tips (Holz et al., 2018; Peters & Long, 1988), and c) more microbes associate with tips compared with other root sections (DeAngelis et al., 2008). Few studies exist investigating spatial patterning of exudation, but some examples suggest that other tissues besides root tips might be involved in exudation. For example, the localization of the malate transporter ALMT1 in Arabidopsis is confined to the root tip in untreated roots, but expands to the entire root system when treated with an activator, aluminum (Kobayashi et al., 2007). This suggests differential malate exudation from different parts of the root, depending on the environment. Similarly, strigolactone exudation is environment‐dependent, with its transporter PDR1 expressed in single cells (hypodermal passage cells) along most of the roots (Kretzschmar et al., 2012). In addition, microbes do not only colonize root tips, but also prominently sites of lateral root emergence, and are found throughout the root system of plants (DeAngelis et al., 2008; Massalha, Korenblum, Malitsky, Shapiro, & Aharoni, 2017). Distinct microbial populations, associated with *B. distachyon* seminal and nodal roots, as well as for nodal root tips versus nodal root bases (Kawasaki et al., 2016), could be influenced by differential exudation by these organs.

We used mass spectrometry imaging to investigate exudation across roots. These data cannot directly be compared to the root morphology and LC/MS data for technical reasons (see Results) and the fact that the exudates were collected from three‐week‐old plants, whereas the imaging experiment was performed with seedlings due to technical limitations. Some ions were observed to be most abundant around the root tip, whereas others were also found in the root elongation and maturation zone, or all along the root axis. In addition, some ions were detected on the root itself, which could mean that they are part of the cell wall, or that they have a low diffusion speed. Despite these limitations, our data suggest that root exudation is a spatially complex process. Exudation might take place in different ways: Root tip‐exuded metabolites might diffuse, due to the absence of Casparian strips or secondary cell walls, or might be actively transported. Metabolites exuded from older root tissues are more likely to be transported, either by channels facilitating diffusion, or by active transport proteins. Future studies are needed to investigate the role of various root zones in exudation to determine which tissues are involved in exudation of various compounds and if exudation differs between root types.

### 3.3 Root exudation per root fresh weight is largely independent of substrate particle size

Root exudate metabolite profiles were unaltered when plants were grown in different particle sizes. As the root weight, root number, and root length correlated and the exudation of compounds was spatially complex, we conclude that exudation profiles are similar across different root morphologies. However, these investigations were limited and may be better informed by comparison of exudation profiles in plants with more radically altered root morphologies, in plants without secondary roots or root hairs.

Exudation profiles were also comparable between plants grown in clay, sand, or glass beads, when collected in vitro. This suggests that the physiochemical environment does not alter plant metabolism, as long as other factors such as nutrient levels, light intensity, and humidity, remain unchanged. However, the exudate metabolite profile of clay‐ versus sand‐ or glass bead‐grown plants was clearly different for in situ exudates (Figure 4). A recent study found differences in sorghum exudates of plants grown in clay, sand, and soil (Miller, Heuberger, Broeckling, & Jahn, 2019). In this study, exudates were collected from roots with rhizosphere substrate still attached. The largest difference in this dataset was observed between soil‐grown and sand‐ or clay‐grown plants, which might be explained by soil‐derived metabolites co‐extracted with root exudates (Miller et al., 2019; Sasse et al., 2018). The authors showed some ions to be specifically up‐ or down‐regulated in exudates of clay‐ versus sand‐grown plants, but their effect was not strong enough to separate the two conditions in a principal component analysis (Miller et al., 2019). This may be explained by their exudate collection method, which was a mixture between the in situ and in vitro conditions utilized here.

Recently, it was suggested that root tips might detect the concentration of rhizosphere metabolites, altering root morphology and exudation accordingly (Canarini, Kaiser, Merchant, Richter, & Wanek, 2019). Thus, clay‐grown plants should exhibit an altered root morphology compared to hydroponically grown plants, as clay sorbs a significant amount of exudates, changing the metabolite concentration around the root tip. However, the root morphology of clay‐grown plants is statistically not different from hydroponically grown plants (Figure 2). Only one particle size of clay was used here, and diffusion rates were lower in this environment. In systems with low diffusion rates, exudate concentration is likely higher around the roots, which might lead to higher exudate re‐uptake than in systems with larger particle sizes (Sasse et al., 2018; Sherson, Hemmann, & Wallace, 2000). Clays with different particle sizes might provoke a root morphology and exudation profile distinct from glass bead‐grown plants and is worth further investigation. Further, substrate particle size might be a factor defining the amount of exudates present in soils.

### 3.4 Exudates are strongly sorbed to clay

The largest difference in exudate profiles observed was between in situ clay‐grown plants and other in situ conditions. Notably, the distinct exudation of clay‐grown plants disappeared when exudates were collected in vitro, indicating that the differences observed resulted from the presence of clay, and not from an altered exudation of compounds by *B. distachyon*.

About 20% of compounds were distinct between hydroponic and clay exudates, and most of these compounds were reduced in abundance in the presence of clay. Among these compounds were organic acids, amino acids, and nucleosides. When clay was incubated with a defined medium, 75% of compounds were reduced in abundance, among them negatively and positively charged compounds, as well as neutral compounds. The higher metabolite retention by clay in the defined medium experiment compared with the plant experiment might be due to several factors: The clay was incubated for two hours with the defined medium, but for three weeks with plants producing exudates. Although exudates were also collected for two hours in the plant experiment, the clay was likely already saturated to some degree with exudates. The quantification of exudate amounts at different plant developmental stages in future studies would enable a better estimation of the total amount of compounds exuded and would correct for the difference in the two experimental setups.

The reduction of metabolite abundance in the presence of clay is most likely due to its high ion exchange capacity, compared with quartz‐based particles such as sand or glass beads (Kabata‐Pendias, 2004). Previous studies investigating sorption of bacterial lysates to ferrihydrite found a depletion of more than half of the metabolites (Swenson, Bowen, et al., 2015). Similarly, incubation of bacterial lysates with a soil consisting of 51% sand, 28% silt, and 21% clay resulted in low metabolite recovery rates (Swenson, Jenkins, Bowen, & Northen, 2015b). These findings are consistent with our data.

Interestingly, two nitrogenous metabolites were higher in abundance in exudates of in situ clay‐grown plants, (Figure 5). These compounds were not detected in clay negative controls, or in in vitro exudates of clay‐grown plants, making it likely that the presence of plants leads to the release of these compounds from clay. Multiple examples exist in literature that describe a release of compounds from minerals by specific exudates. For example, plant‐derived organic acids such as malate and citrate solubilize mineral‐bound phosphate (Neumann & Martinoia, 2002), and plant‐derived oxalate releases organic compounds bound to minerals, making them available to microbial metabolism (Keiluweit et al., 2015).

Altered exudation depending on the growth substrate was also described for tomato, cucumber, and sweet pepper growing in stone wool, with higher exuded levels of organic acids and sugars compared with glass bead‐grown plants (Kamilova et al., 2006). The authors suggest that the presence of aluminum ions in stone wool might be responsible for the altered exudation observed. As the authors did not investigate in vitro‐collected exudates of stone wool‐grown plants, it is unclear to which degree the observed effect was due to changes in plant metabolism or due to the presence of stone wool.

In soils, metabolite sorption to minerals can lower decomposition rates (Baldock & Skjemstad, 2000). Also, the amount of clay in soil is correlated with retention of labeled carbon in soils (Baldock & Skjemstad, 2000). In clay‐dominated soils, the size of clay particles shapes how much carbon can be retained: large clay aggregates were found to adsorb more carbon than smaller aggregates (Six & Paustian, 2014). Here, we only investigated one size of clay particles. Thus, it would be prudent to investigate the sorption behavior of clays with different particle sizes, and the ability of microbes to subsequently desorb these compounds. In natural systems, the presence of large amounts of clay with a specific particle size likely results in the sorption of plant‐derived compounds to particles, changing the direct availability of these compounds to heterotroph organisms and, thus, altering soil processes.

### 3.5 Microbial desorption of clay‐bound metabolites

Microbes can release sorbed compounds from minerals, and they likely preferentially colonize minerals that are associated with compounds missing from the environment (Uroz et al., 2015). The rhizobacterium *Pseudomonas fluorescens* utilized in this study was indeed able to desorb metabolites from clay, utilizing them as a carbon source for growth (Figure 6). In soils, root exudation creates zones with high metabolite concentrations. The released exudates can either be directly taken up by root‐associated microbes or sorbed to minerals. Although *P. fluorescens* was able to grow on particles conditioned with exudates, it did not grow on the effluent of the washed particles. This suggests that the organism is able to release mineral‐bound metabolites as an additional source for growth—a trait that supports competitiveness and survival in the rhizosphere. Root‐associated bacteria have distinct exudate substrate preferences from bulk soil bacteria (Zhalnina et al., 2018), which might also define the kind of compounds bacteria are able to release from minerals (Uroz et al., 2015). Our results are further evidence that minerals play an important role in plant–microbe interactions by sorbing root exudates, which can later be solubilized by microbes for growth.

We conclude that alteration in particle size affects root morphology in *B. distachyon.* Root exudation was constant per root fresh weight, and the exudate metabolite profiles were similar across root morphologies. Mass spectrometry imaging detected ion abundances across various regions of the root system, suggesting involvement of different tissues in exudation. Exudates were strongly sorbed by clay, significantly reducing the availability of free metabolites. Some of the clay‐bound metabolites however could be utilized by a rhizobacterium for growth. Soil clay content thus is likely an important factor to consider when investigating root exudates or plant–microbe interactions in natural environments.

## 4 MATERIALS and METHODS

### 4.1 Substrates and particle sizes

Substrates with various particle sizes and surface chemistries were chosen as experimental systems to assess changes in root morphology and exudation. The particle sizes used correspond to large soil particles (>2 mm), intermediary particles (53–250 µm), and small particles (<53 µm)(Six et al., 2000). Glass beads constitute an inert experimental system, for which the diameter of the spheres is defined, and the particles have a defined mineral composition (sizes: 3, 2, 1, 0.5 mm, Sigma‐Aldrich 1,040,150,500, 18,406, Z250473, Z250465). The sand and clay substrates constitute more natural environments than glass beads (sand sizes: 4 mm, 250 µm, 5 µm; clay size: 4 mm. Sand 4 mm: Rock‐It Direct LLC, 612 Silica Sand; Sand 250 µm: Sigma‐Aldrich SiO_2_274739; Sand 5 µm: Sigma‐Aldrich SiO_2_ S5631; Clay: Turface Athletics, MVP Calcined Clay). The sand and clay mineral composition was either defined by the manufacturer or determined here. The manufacturer's specifications for the clay are as follows: calcined, non‐swelling illite clay with 60% minimum amorphous silica (SiO_2_), 5% Fe_2_O_3_, and <5% Al_2_O_3_, CaO, MgO, K_2_O, Na_2_O, and TiO_2_, approximately 41% 2.38 mm (mesh 8), 16% 3.36 mm (mesh 6), 24% 1.68 mm (mesh 12), 18% 0.84 mm (mesh 20), and 1% <0.84 mm (mesh 30 and smaller), and pH 6.5 ± 1.

All substrates were acid‐washed with 0.1 M HCl for 1 hr at 200 rpm, rinsed five times with MilliQ water, and baked for 30 min at 200°C.

### 4.2 Sorption test with defined medium

To assess the chemical properties of the substrates, the sorption of metabolites to the substrates was assessed by incubating them with a defined medium (Figure 1). The defined medium consisted of amino acids, organic acids, sugars, nucleobases, nucleosides, and others, see Table S2) (Jenkins et al., 2017). The various substrates were sterilized, and the defined medium was prepared as a sterile, 20 µM equimolar solution. The substrates were fully submerged in the defined medium in sterile conditions and incubated at 24°C for 8 hr. The sterility of the system was confirmed by plating an aliquot on LB plates, followed by a 3‐day incubation. The defined medium was removed by pipetting. The recovered volume was recorded; for substrates with smaller particle sizes, the entire volume could not be reclaimed. Samples were filtered through a 0.45‐µm filter (4,654, PALL Life Sciences) and frozen at −80°C. See “Liquid chromatography–mass spectrometry sample preparation” for sample processing.

### 4.3 Sample preparation for liquid chromatography–mass spectrometry

The frozen samples were lyophilized (Labconco FreeZone lyophilizer), resuspended in 3 ml LC/MS grade methanol (CAS 67–56–1, Honeywell Burdick & Jackson, Morristown, NJ), vortexed three times for 10 s, sonicated for 20 min in a water bath at 24°C, and incubated at 4°C for 16 hr for salt precipitation. Samples were then centrifuged for 5 min at 5,000 g and 4°C, and supernatants were transferred to new microcentrifuge tubes and evaporated at 24°C under vacuum until dry. The dried extracts were resuspended in 500 µl LC/MS grade methanol, and the above procedure centrifugation, drying and resuspension procedure were repeated. Finally, samples were resuspended in 100% LC/MS grade methanol with 15 µM internal standards (767,964, Sigma‐Aldrich), with the solvent volume being proportional to the root biomass (0.77 g/ml) (Sasse et al., 2018).

### 4.4 Diffusion experiment

In the various experimental systems used here, exudation rates could be limited by diffusion. To determine the diffusion rates of various substrates, sterilized substrates (3‐mm, 2‐mm, 1‐mm, 0.5‐mm glass beads, 4‐mm sand, 4 mm clay, or no substrate) were added to pipettes with a 50 ml volume. The pipettes were sealed at the bottom with parafilm, placed vertically, 50 ml of substrate was added, and approximately 25 ml of sterilized 0.5× MS was added to fully immerse submerse the substrate (there was no correlation between the amount of 0.5× MS added and the volume needed to immerse the substrate). The experimental setup was sterile, but the experiment was conducted in non‐sterile conditions. Congo red 4B (C6277, Sigma‐Aldrich) was solubilized in water at a concentration of 20 mg/ml, and 250 µl of the dye was added simultaneously to pipettes containing the various substrates. The front of the dye was followed recorded over time up to 4.5 hr. Initially, the movement of the dye front was supported by mass flow (gravity).

### 4.5 Plant growth conditions

*Brachypodium distachyon* Bd21‐3 seeds were dehusked and sterilized in 70% v/v ethanol for 30 s, and in 6% v/v NaOCl with 0.1% v/v Triton X‐100 for 5 min, followed by five wash steps in water. Seedlings were germinated on 0.5× Murashige & Skoog plates (0.5× MS basal salt agar: MSP01, Caisson Laboratories; 6% w/v Bioworld Phytoagar, 401,000,721, Fisher Scientific, pH 5.7) in a 150 µmol/m^2^ s^−1^ 16‐hr light/8‐hr dark regime at 24°C for three days.

Weck jars (743; Glashaus Inc.) were rinsed five times with MilliQ water, sprayed with 70% v/v ethanol, treated with UV light for 1 hr in a laminar flow hood, and dried over night. The jars were filled with 150 ml of the respective substrate, and 50 ml of 0.5× MS basal salts liquid medium. Three seedlings were transferred into each jar, with the roots buried in the substrate. As a control, jars without substrate (0 mm, hydroponic control) were prepared: PTFE mesh (1100T45; McMaster Carr) was cut to fit the size of the jar, and autoclaved. Three openings were cut into the mesh to hold the seedlings. The mesh was transferred to jars with 50 ml 0.5× MS medium. For each condition, an experimental negative control was prepared containing substrate, but no seedlings. The experimental control jars were treated the same as the jars containing plants. To enable gas exchange, two strips of micropore tape (56222‐182, VWR) were placed across the jar opening, and the lid was set on top and wrapped with micropore tape (56222‐110, VWR) to ensure sterility.

Plants were grown in a 16‐hr light/8‐hr dark regime at 24°C with 150 µmol/m^2^ s^−1^ illumination, and the growth medium was replaced weekly: The old medium was removed by pipetting, and new 0.5× MS was added. Sterility of the jars was tested in week 3 by plating 50 µl of medium on Luria‐Bertani (LB) plates, following by three days incubation at 24°C. Plants were grown for 21 days before exudate collection and root morphology determination.

### 4.6 Root exudate collection

To distinguish the effect of particle chemistry and altered plant metabolism, exudates were collected in two steps: First, exudates were collected from plants growing in the respective substrates (“in situ*”*), and second, plants were removed from the substrate to collect exudates in a liquid medium (“in vitro*”*). In detail: At 21 days after germination (21 dag), the medium in the jars was exchanged, and jars were incubated at 24°C with 150 µmol/m^2^ s^−1^ illumination for 2 hr (“in situ*”* treatment). Subsequently, plants were carefully removed from the substrate, and the roots of plants originating from the same jar were incubated submerged in 50 ml 0.5× MS for 2 hr (“in vitro*”* treatment) to account for the effect of the substrate presence on exudation (Figure S1). Root exudates were passed through a 0.45‐µm filter (4,654, PALL Life Sciences), and frozen at −80°C. See “Liquid chromatography–mass spectrometry sample preparation” for sample processing.

### 4.7 Root morphology imaging and analysis

After exudate collection, root systems were arranged on a glass plate and imaged, and root and shoot fresh weight was recorded. The root systems were traced with the SmartRoot plugin (version 4.21) in ImageJ (version 2.0.0) (Lobet, Pagès, & Draye, 2011), and roots were assigned as first order (primary and crown roots), second order (lateral roots of primary and crown roots), and higher order (laterals of secondary roots). For each condition, a minimum of five root systems was analyzed.

Correlation analysis of root parameters was calculated using Excel's Pearson correlation coefficient (PCC) function, and the t‐value was calculated using the following formula (*n* is the number of observations):

$$t=\frac{\text{PCC}\sqrt{n-2}}{\sqrt{1-{\text{PCC}}^{2}}}$$

### 4.8 MALDI Mass Spectrometry Imaging

Mass spectrometry imaging was used to investigate spatial patterning of root exudation across the root system. *Brachypodium distachyon* seeds were sterilized and germinated on 0.5 MS plates as described above. A stainless steel MALDI plate was cleaned with 100% v/v ethanol, and a 7 × 7 cm square of aluminum foil was affixed to the plate with double‐sided scotch tape. The foil was overlayed with 4 ml 0.1% ultrapure agarose (LS16500, Invitrogen, Toronto, ON, CA) to create a thin layer of agarose. Four‐day‐old seedlings were transferred to the agarose layer, and gentle pressure was applied with a spatula to embed the roots in the agarose. The stainless steel plate was transferred into a petri dish plate to keep humidity constant and incubated for 6 hr in a growth chamber with 150 µmol/m^2^ s^−1^ illumination and 24°C.

MALDI matrix was prepared as follows: 10 mg/ml a‐cyano‐4‐hydroxycinnamic acid (70,990, Sigma‐Aldrich, St. Louis, MO, USA) and 10 mg/ml Super‐DHB (50,862, Sigma‐Aldrich, St. Louis, MO, USA) were dissolved in 75% v/v methanol, 24.9% LCMS‐grade water, and 0.1% formic acid. The plate with the seedlings was removed from the growth chamber, leaves were cut to ensure flatness of the sample, and the sample was sprayed with MALDI matrix, which simultaneously desiccated the tissue. The plate was incubated for 24 hr in a vacuum desiccator until completely dry. Mass Spectrometry Imaging was performed using a 5,800 MALDI TOF/TOF (AbSciex, Foster City, CA, USA) in positive reflector MS mode with an Nd:YAG laser (200 Hz, 4,400 laser intensity) acquiring spectra over a range of 50−2000 Da (1,000 Da focus mass) and accumulating 20 shots/spot. The 4,800 Imaging Tool software (Novartis and Applied Biosystems) was used to raster across the sample and record spectra in x‐y step‐sizes of 75 × 75 μm. Data viewing and image reconstruction were performed using OpenMSI (https://openmsi.nersc.gov) (Rübel et al., 2013).

### 4.9 Liquid chromatography–mass spectrometry methods and analysis

Metabolites were chromatographically separated with a hydrophilic liquid interaction chromatography on an InfinityLab Poroshell 120 HILIC‐z 2.7 µm, 2.1 mm × 150 mm (Ag683775‐924; Agilent Technologies) and detected with a Q Exactive Hybrid Quadrupole‐Orbitrap Mass Spectrometer equipped (Thermo Fisher Scientific). Chromatographic separations were performed on an Agilent 1,290 series HPLC system with the following parameters: The column temperature was held at 40°C, the sample injection volume was 3 µl, and the autosampler was set at 4°C. A gradient of mobile phase A (5 mM ammonium acetate, 0.2% acetic acid, 5 µM methylene‐di‐phosphonic acid in water) and B (5 mM ammonium acetate, 0.2% acetic acid, 95% v/v acetonitrile in water) was used for metabolite retention and elution as follows: column equilibration at 0.45 ml/min in 100% B for 1.0 min, a linear gradient at 0.45 ml/min to 11% A over 10 min, a linear gradient to 30% A over 4.75 min, a linear gradient to 80% A over 0.5 min, hold at 0.450 ml/min and 80% A for 2.25 min followed by a linear gradient to 100% B over 0.1 min, and re‐equilibration for an additional 2.4 min. Each sample was injected twice: once for analysis in positive ion mode and once for analysis in negative ion mode. The mass spectrometer source was set with a sheath gas flow of 55, aux gas flow of 20 and sweep gas flow of 2 (arbitrary units), spray voltage of |±3| kV, and capillary temperature of 400°C. Ion detection was performed using the data‐dependent MS2 Top2 method, with the two highest abundance precursory ions (1.0 m/z isolation window, 17,500 resolution, 1e5 AGC target, stepped normalized collisions energies of 10, 20 and 40 eV) selected from a full MS pre‐scan (70–1050 m/z, 70,000 resolution, 3e6 AGC target, 100 ms maximum ion transmission) with dd settings at 1e3 minimum AGC target, charges excluded above |8|, and a 7 s dynamic exclusion window. Internal and external standards were included for quality control purposes, with blank injections between every unique sample, and samples were injected in randomized order. Raw data files and results have been deposited in the online Joint Genome Institute Genome Portal at: https://genome.jgi.doe.gov/portal/201Rootabolomics_FD/201Rootabolomics_FD.info.html under IDs FD 1,266,726 and SP 1,266,729.

### 4.10 Metabolite identification and statistical analysis

Ion chromatograms corresponding to metabolites represented within our in‐house standard library were extracted from LC/MS data with Metabolite Atlas (https://github.com/biorack/metatlas) (Bowen & Northen, 2010; Yao et al., 2015). Metabolites were identified following the Metabolomics Standards Initiative conventions, using the highest confidence level (“level 1,” MSI identifications), which is identified as at least two orthogonal measures versus authentic chemical standards (Sumner et al., 2007). Three orthogonal measures were used to compare samples with authentic chemical reference standards: retention time (within 0.5 min versus standard), fragmentation spectra (manual inspection), and accurate mass (10 ppm in positive mode and 20 ppm in negative mode). Peak height and retention time consistency for the LC/MS run were ascertained by analyzing quality control samples that were included at the beginning, during, and at the end of the run. Internal standards were used to assess sample‐to‐sample consistency for peak area and retention times.

Metabolite peak heights were scaled relative to the maximum peak height in any sample within an experiment to allow for relative comparison of peak heights between samples (e.g., if a compound of interest is significantly different between samples), but not for absolute metabolite level quantification (e.g., µg of a compound of interest per gram tissue). Chemical classes were assigned to metabolites with the ClassyFire compound classification system (Djoumbou Feunang et al., 2016).

To explore the variation between experimental conditions, the metabolite profiles were PCA‐ordinated, and the 95% confidence level was displayed as ellipses for each treatment. Hierarchical clustering analysis with a Bray–Curtis Dissimilarity Matrix was performed with the python 2.7 Seaborn package. Metabolite significance levels were analyzed with the Python SciPy ANOVA test coupled to a python Tukey's honestly significant difference test with alpha = .05 corresponding to a 95% confidence level.

### 4.11 Rhizobacterium growth experiment

To test whether metabolites sorbed to clay were accessible for a plant‐associated bacterium, the desorption rates of metabolites from different substrates were tested in a first experiment, and the growth rate of *Pseudomonas fluorescens* WCS415 on various substrates pre‐incubated with metabolites was tested in a second experiment.

The desorption rate of metabolites from substrates was determined for glass beads (0.5–3 mm), sand (5 µm‐4 mm), and clay (4 mm). The substrates were incubated with 50 times concentrated defined medium (50× DM, see: “Sorption test with defined medium” section, (Jenkins et al., 2017)) or with 0× DM (DM without carbon sources, but with vitamins and minerals) for 6 hr at 23°C. The substrates were subsequently washed three times with water, to remove soluble metabolites. The recovered metabolites of all three steps were analyzed by LC/MS, as described above.

Substrates were added to a 12‐well plate (2 cm^3^ each), and 2 ml of 0× DM was added to each well. A *Pseudomonas fluorescens* WCS415 preculture was grown in 5 ml 20× DM for 16 hr at 30°C, 200 rpm. The culture was pelleted at 4,000 g, 23°C for 5 min, and resuspended in 0× DM. The wells were inoculated with an initial optical density (determined at 600 nm) of 0.05 in triplicates. The plates were incubated at 30°C for 3 d (no shaking), 1 ml of the supernatant was removed to determine OD at 600 nm. Positive growth controls were *P. fluorescens* grown in the same experimental setup in 50×, 20×, 10×, and 0× DM, but without substrate. A set of negative controls was prepared to account for different variables in the experiment: Substrates incubated with 0× DM with bacteria were set up as a growth control, accounting for metabolites already adsorbed to clay. Substrates incubated with 50× and 0× DM but without bacteria were used to control for changes in optical density of clay caused by DM.

The metabolite desorption experiment was performed by adding 2 cm^3^ of clay pre‐incubated with 50× or 0× DM to a 12‐well plate in triplicates, followed by the addition of 2 ml of 0× DM. The plate was incubated for 3 d at 30°C. Subsequently, 1.5 ml of the supernatant was removed by pipetting and placed in a new 12‐well plate. Half of the wells were inoculated with *P. fluorescens,* the other half served as negative controls. OD at 600 nm was determined after 3 d of growth at 30°C.

## AUTHOR CONTRIBUTIONS

J.S. and T.R.N. designed the project and experiments; J.S. supervised the experiments; J.S., S.M.K. M.d.R., and K.Z. performed the experiments; J.S., S.M.K., J.J, and K.W. performed data analysis; J.S. and T.R.N. wrote the manuscript with input from all authors. J.S. and T.R.N. agree to be responsible for contact and ensure communication.

## Supporting information

Supplementary Material 1

Table S2

Supplementary Material 2
