# Supplementary material for: Root morphology and exudate availability are shaped by particle size and chemistry in Brachypodium distachyon
Source: Plant Direct. 2020 Jul 2;4(7):e00207. doi: 10.1002/pld3.207 (PMC7330624; doi:10.1002/pld3.207)
Supplement: Supplementary file 1 — Supplementary Material 1 [file PLD3-4-e00207-s001.pdf]

Figure S1: Experimental setup

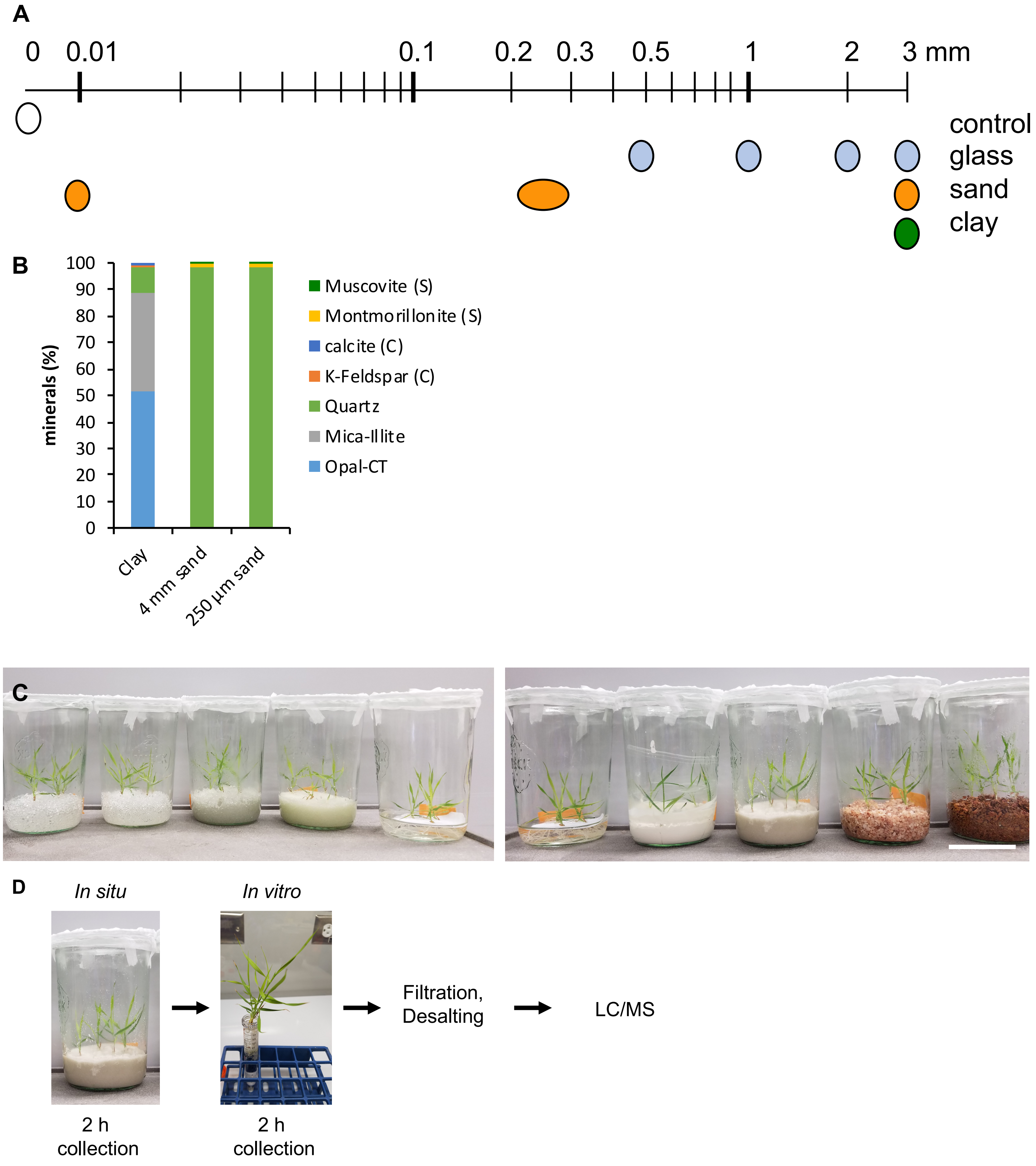

Figure S1 | Experimental setup (A) Illustration of particle sizes utilized, logarithmic scale. **(B)** X-ray diffraction analysis of 4 mm clay, 4 mm sand, and 250 µm sand. Trace elements are indicated as being present in sand (S) or clay (C). **(C)** Jar setup, one jar per condition is depicted. Top panel, from left: 3 mm, 2 mm, 1 mm, 0.5 mm glass beads, hydroponic control. Bottom panel, from left: hydroponic control, 5 µm sand, 250 µm sand, 4 mm sand, 4 mm clay. Scale bar: 10 cm. **(D)** Root exudate collection procedure, the in vitro picture displayed is representative of the setup, but a larger tube (50 ml) was used for exudate collection.

Figure S2: Additional root morphology data

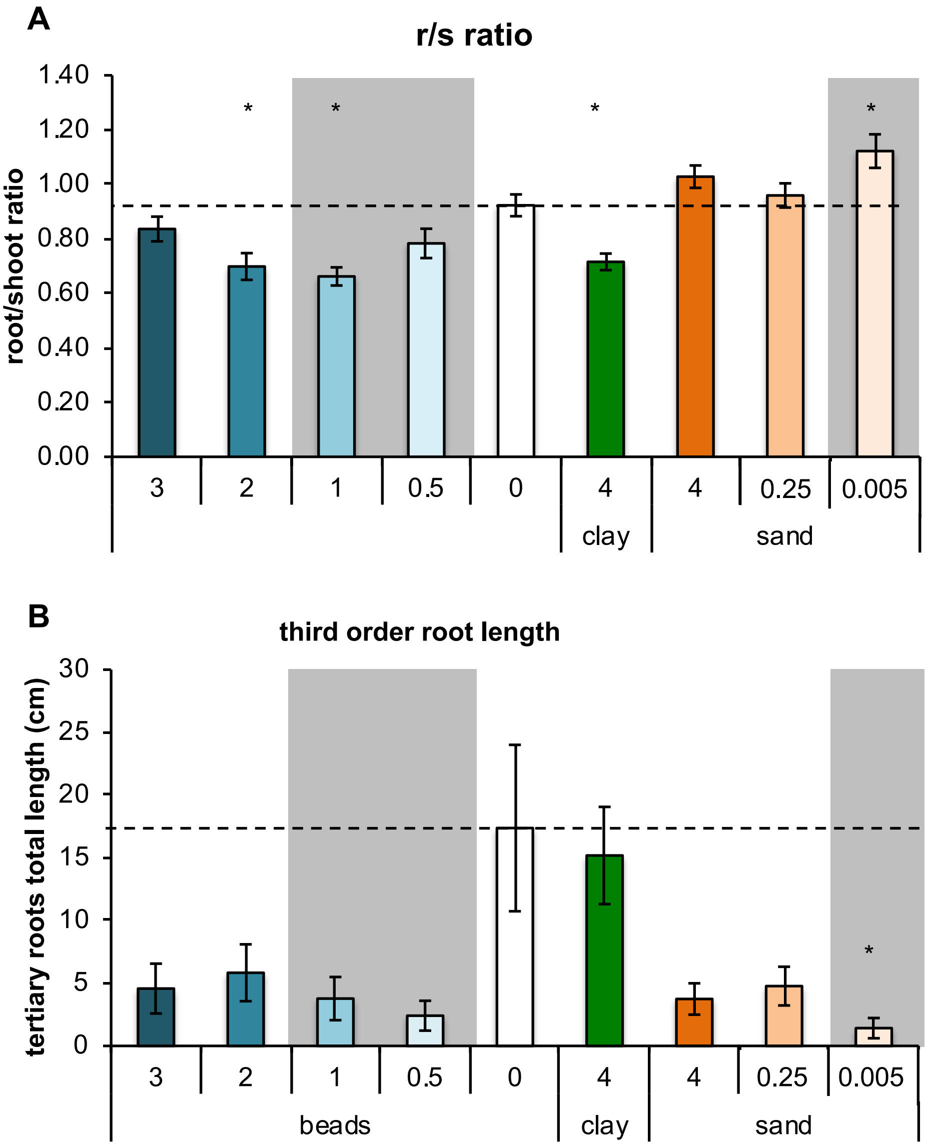

Figure S2 | Additional root morphology Root/shoot ratio **(A),** third order root length **(B)** first order **(C)**, second order **(D)**, and third order **(E)** root numbers. Data are means ± SEM, n>5. Significant differences are displayed as asterisks (*, p = 0.05) of substrates compared to hydroponic control (0, dashed line). Tissue weights, total root length and number, first and second order root lengths are given in Fig. 2. Grey areas: plants with weight and root morphology distinct from hydroponic controls.

Figure S2: Additional root morphology data

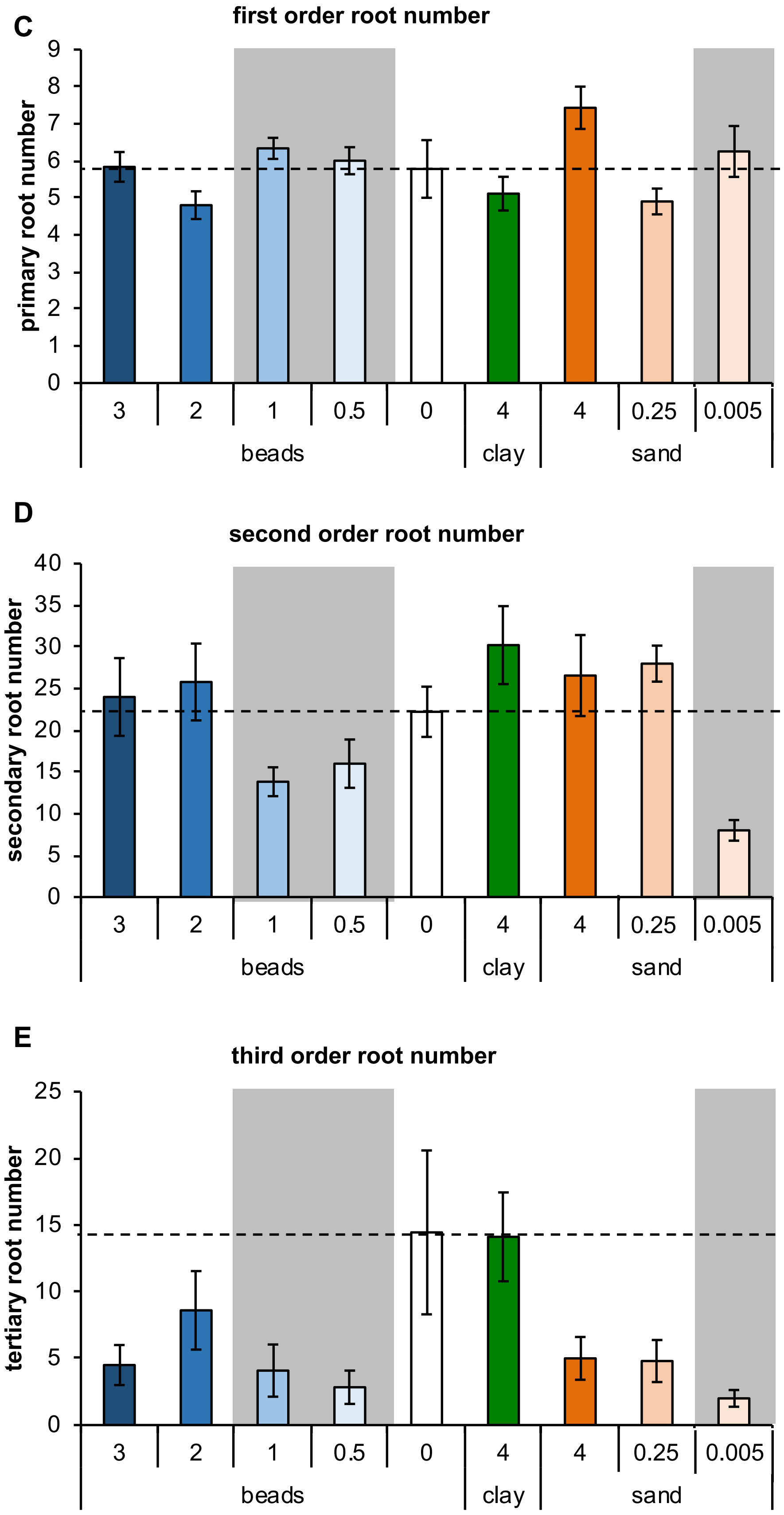

Figure S3: representative root morphologies

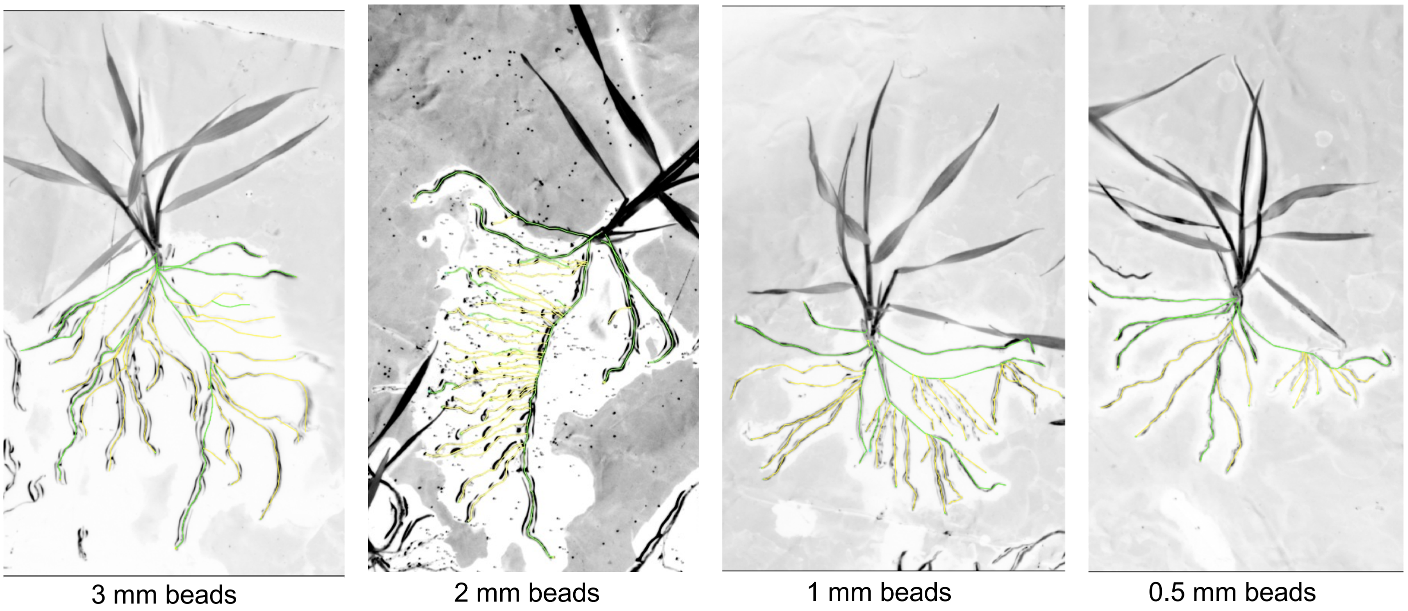

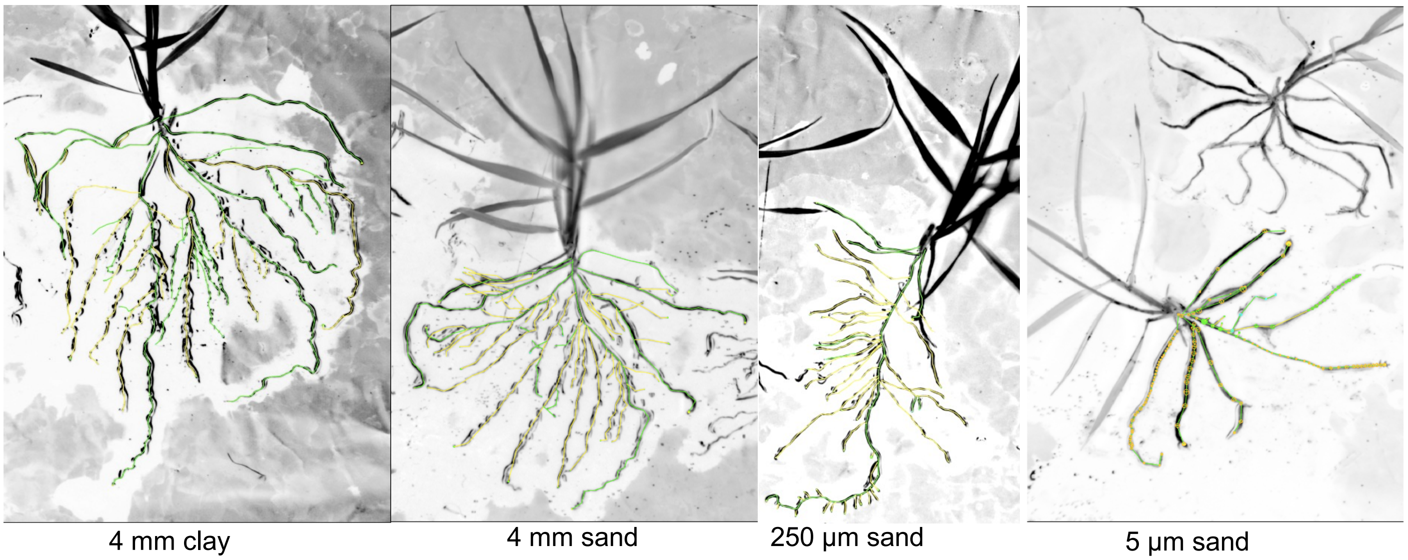

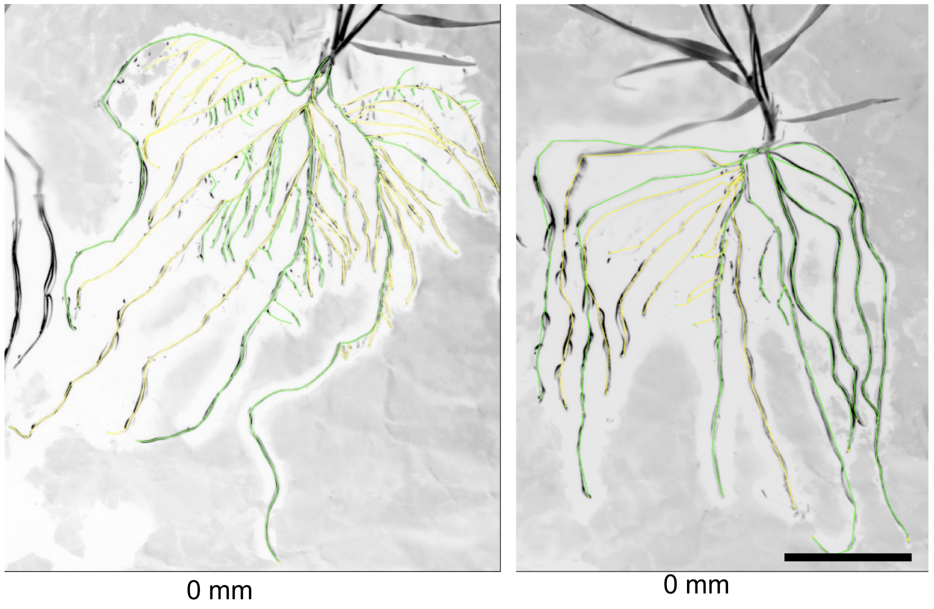

Figure S3 | Representative root morphologies Representative root morphologies of 3-week-old *B. distachyon* grown in the various substrates. First order roots were traced in green, second order roots in yellow, and third order roots in green (SmartRoot, {Lobet:2011fl}). Quantification of root systems are displayed in Fig. 2 and Fig. S2. Scale bar: 3 cm.

Figure S4: Correlation analysis of root parameters

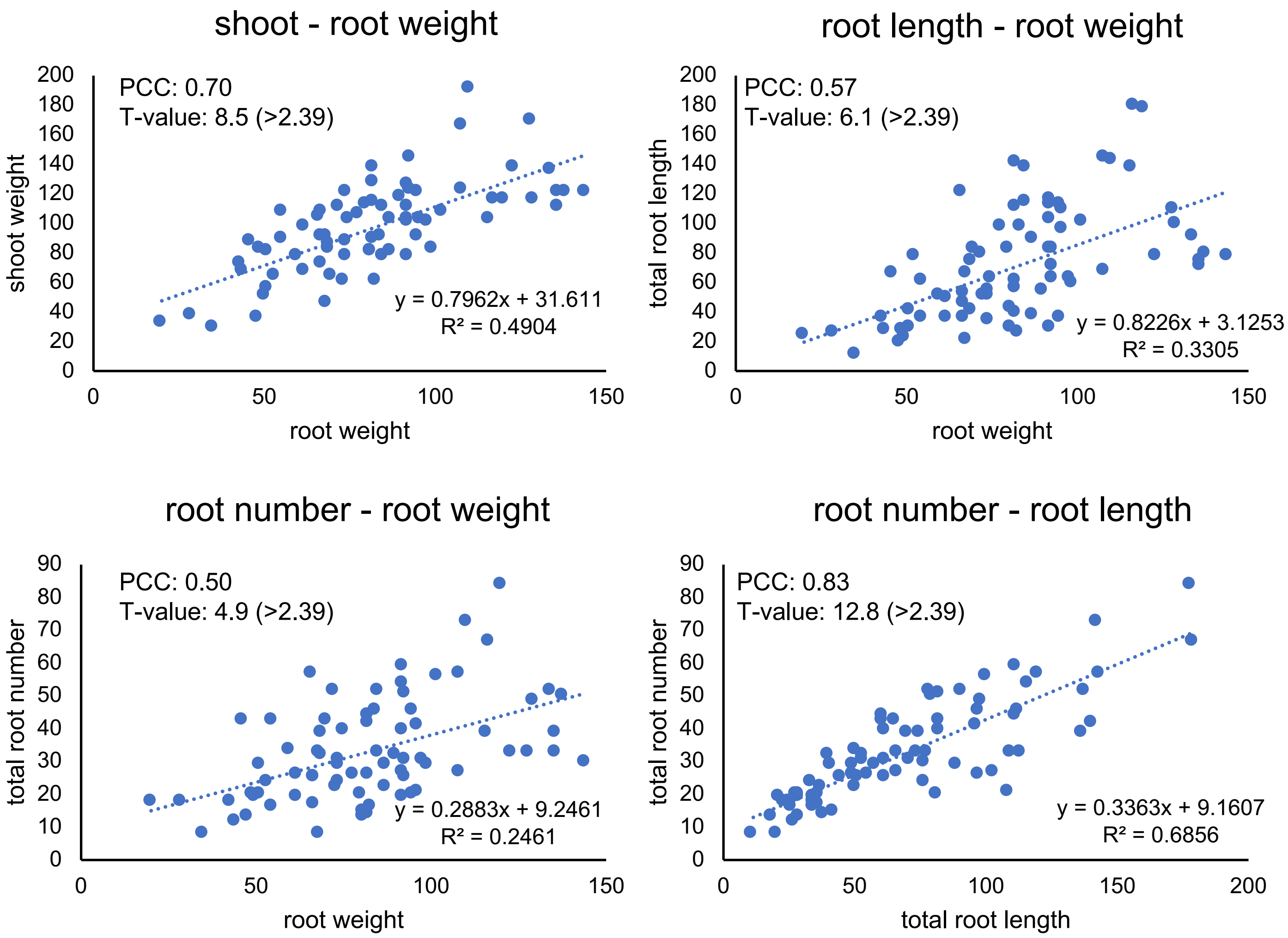

Figure S4 | Correlation analysis of root parameters Correlation plots of various root parameters. Dashed lines are linear regressions, equations and R^2 values are given. Pearson correlation coefficients (PCC) were calculated, and the respective t-values are given.

Figure S5: *B. distachyon* seedling exudation visualization

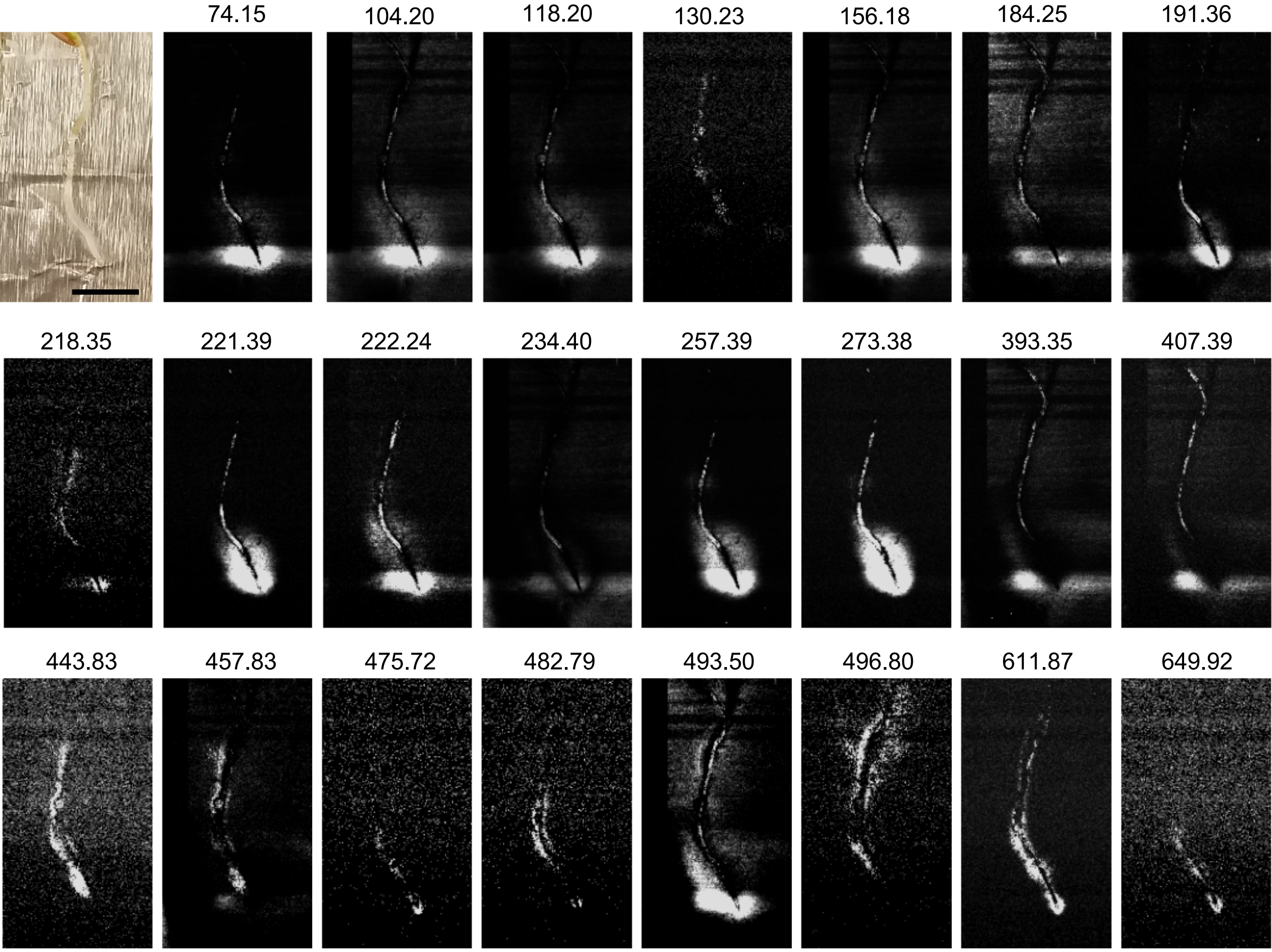

Figure S5 | Additional spatial exudation patterns Plants were incubated for 6 h to allow for exudation. Distinct root-associated patterns of several ions were observed with Mass Spectrometry Imaging. The *m/z* of the different ions observed is indicated above the panels. Selected panels are presented in Fig. 3. Ion 156.18 is possibly a M+K+H adduct of 118.20. Scale bar: 1 cm.

Figure S6: Distinct metabolites in various substrates

A *In situ*

			Glass beads				Sand		
		0 mm	3 mm	2 mm	1 mm	0.5 mm	4 mm	250 µm	5 µm
Glass beads	3 mm	5							
	2 mm	8	4						
	1 mm	14	10	14					
	0.5 mm	10	5	5	5				
Sand	4 mm	8	5	7	4	2			
	250 µm	12	9	18	5	5	5		
	5 µm	14	13	15	8	5	5	4	
Clay	4 mm	23	15	24	8	13	6	3	5

B *In vitro*

			Glass beads				Sand	
		0 mm	3 mm	2 mm	1 mm	0.5 mm	4 mm	250 µm
Glass beads	3 mm	5						
	2 mm	4	0					
	1 mm	5	3	1				
	0.5 mm	4	5	1	0			
Sand	4 mm	3	1	1	0	2		
	250 µm	1	3	3	2	0	0	
Clay	4 mm	9	11	8	12	7	2	1

C Defined medium

			Glass beads		Sand	
		DM	3 mm	1 mm	4 mm	250 µm
Glass beads	3 mm	15				
	1 mm	23	17			
Sand	4 mm	21	16	14		
	250 µm	45	44	37	43	
Clay	4 mm	51	53	48	51	40

Figure S6 | ANOVA-Tukey test results Percent of significantly different metabolites in pairwise comparisons of *in situ* collected exudates **(A)**, *in vitro* collected exudates **(B)**, and substrates incubated with defined medium **(C)**. Data are means of 3 biological replicates, ANOVA, p = 0.05.

Figure S7: Additional analysis exudate metabolic profiles

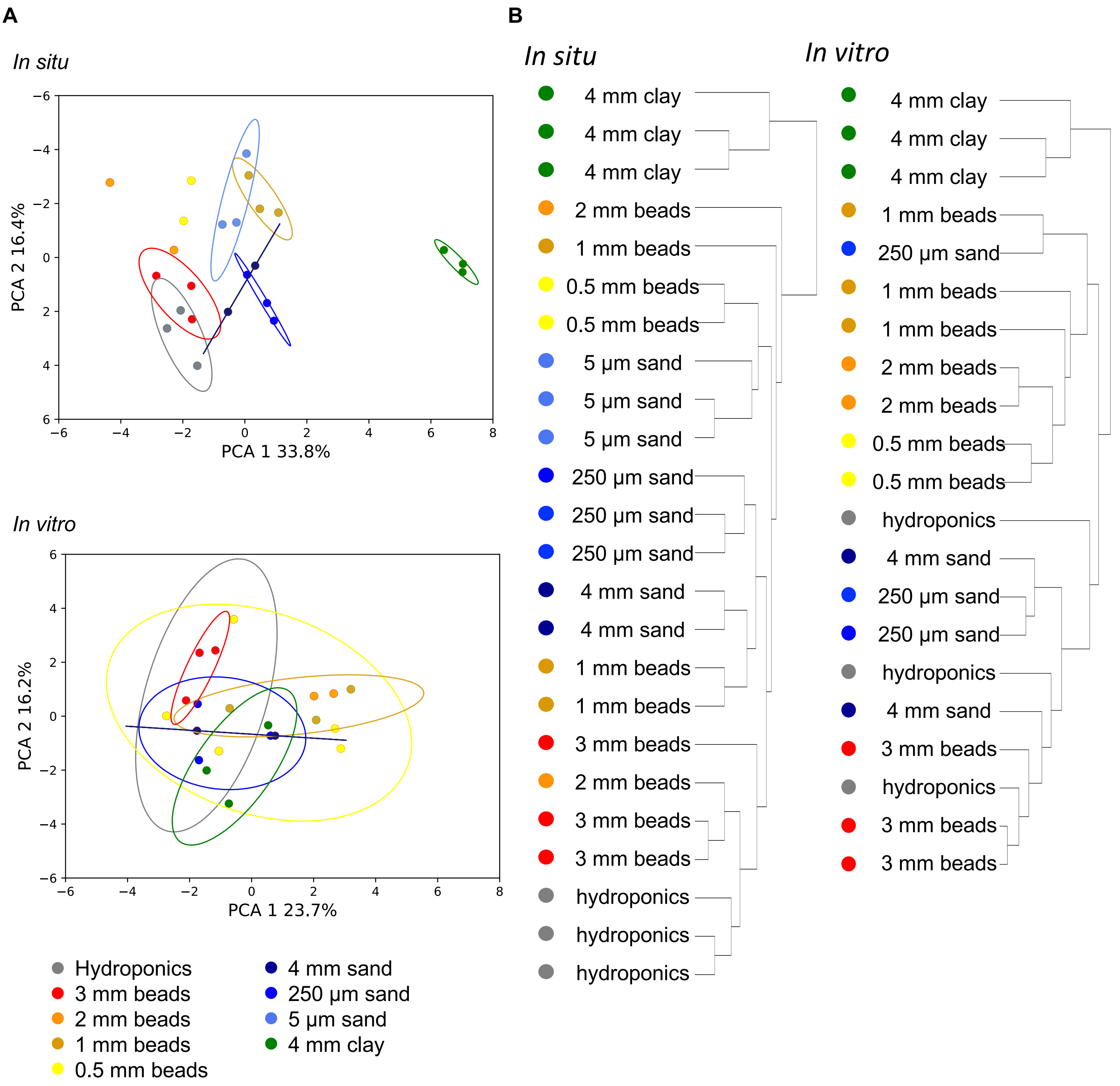

Figure S7 | *In situ* and *in vitro* exudate metabolite profile analyses Principal component (PC) analysis of metabolite profiles of root exudates collected *in situ* or *in vitro* **(A)**, and hierarchical clustering of the same datasets **(B)**. PC analyses of the data grouped by root morphology is displayed in Fig. 4.

Figure S8: Distinct metabolites in grouped in situ substrates

A

	beads big	beads small	sand big	sand small
beads small	**13**			
sand big	13	6		
sand small	11	5	**2**	
hydroponics	7	17	13	15

B

		Glass beads				Sand			Clay
	**0mm**	**3mm**	**2mm**	**1mm**	**.5mm**	**4mm**	**250µ**	**5µ**	**4mm**
2',3'-cyclic AMP	62	84	18	0	0	22	9	7	0
1-Methyladenosine	71	82	68	14	13	25	20	18	3
cytidine 2',3'-cyclic monophosphoric acid	78	77	68	20	27	22	8	2	0
Guanine	44	74	66	21	49	14	17	28	0
Acetyl-hexosamine	74	71	75	31	30	48	21	32	21
Pantothenic acid	36	66	76	40	37	40	19	12	8
Indole-3-acetic acid / isomer	57	62	71	14	7	5	8	7	0
5'-Methylthioadenosine	44	58	91	10	21	21	2	6	0
Cytidine	46	44	84	14	28	36	14	23	0
Caffeic acid	1	8	2	75	7	2	8	21	2
2/3-Hydroxybenzoic acid	4	6	7	51	15	4	6	9	13
Benzoic acid	7	9	12	20	14	6	5	5	5
Trans-Cinnamic acid	6	3	6	18	20	5	3	6	3

C

		Glass beads				Sand			Clay
	**0mm**	**3mm**	**2mm**	**1mm**	**.5mm**	**4mm**	**250µ**	**5µ**	**4mm**
L-Carnitine	8	7	6	9	6	8	11	66	8
Cytosine	6	7	28	13	6	4	17	53	29

Figure S8 | Distinct metabolites in grouped in situ substrates (A) Number of significantly different metabolites in pairwise comparisons of metabolite profiles grouped by root morphology (ANOVA, p = 0.05). Substrates are grouped as follows: beads big: 3 mm, 2 mm glass beads; beads small: 1 mm, 0.5 mm glass beads; sand big: 4 mm, 250 µm sand; sand small: 5 µm sand. Heatmap of metabolite abundances significantly different between in situ collected exudates in big vs small beads (**B**) and big vs small sand (**C**), ANOVA, p = 0.05. Values indicate average peak heights, scaled to the maximum abundance for each compound. All substrates are displayed for comparison. Full dataset is provided in Table S2.

Figure S9: Metabolite recovery from substrates

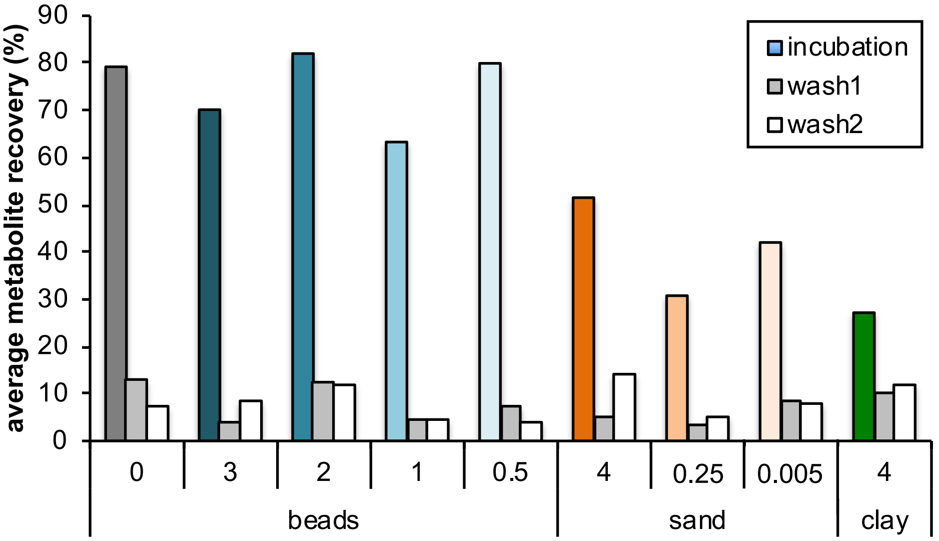

Figure S9 | Metabolite recovery from substrates Relative abundance of defined medium metabolites in percent in various substrates after incubation (colored bars), after a first wash (grey bars), and a second wash (white bars). Data is normalized by metabolite abundance in defined medium incubated without substrate; n=1.

Figure S10: Rhizobacterium utilization of clay sorbed metabolites

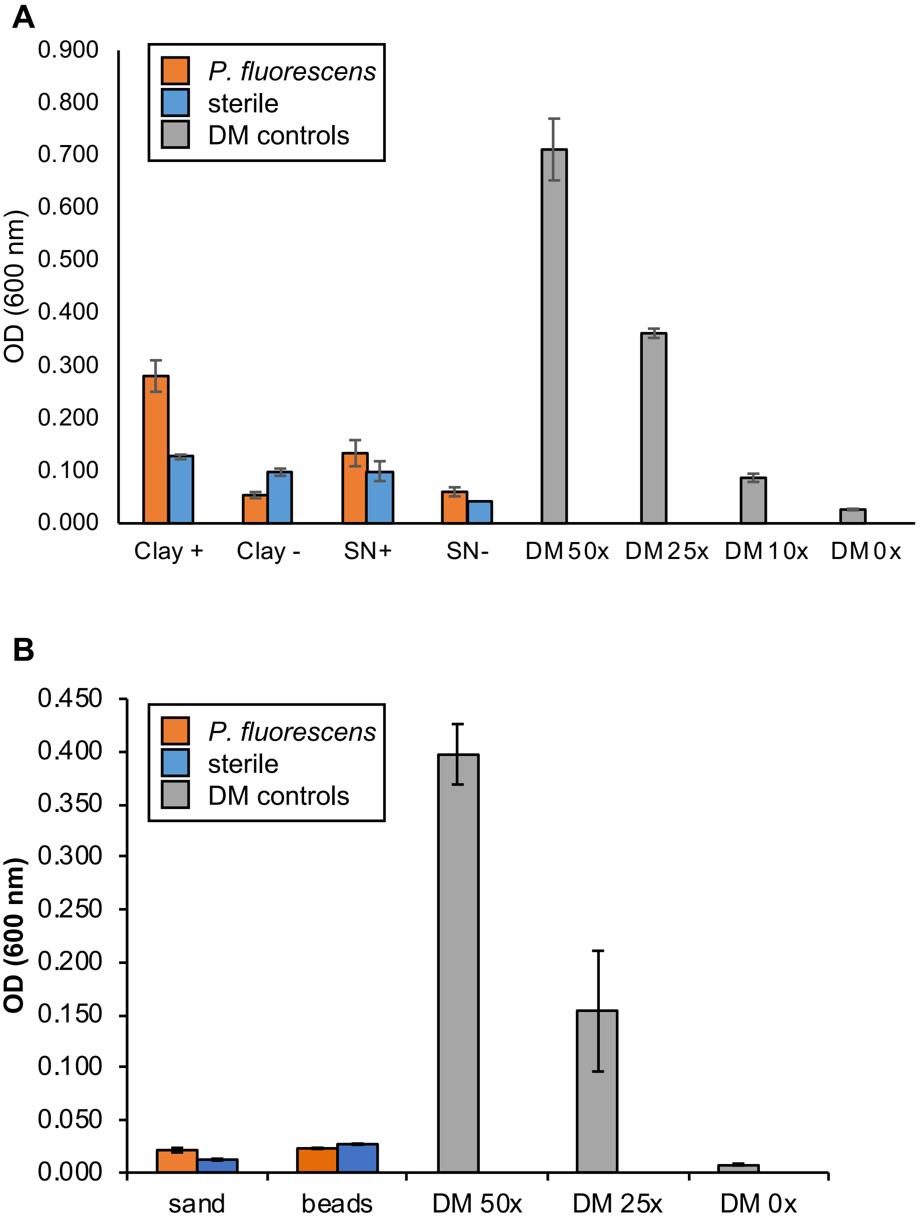

Figure S10 | Rhizobacterium utilization of clay sorbed metabolites (A) Optical density (OD at 600 nm) of *P. fluorescens* (orange bars) grown in clay pre-incubated with 50x defined medium (DM, Clay +) or 0x DM (Clay -), or in supernatant of clay pre-incubated with 50x DM (SN +) or 0x DM (SN -). Sterile controls (blue bars) indicate substrate background (no bacteria control). Data normalized by substrate background is displayed in Fig. 6. **(B)** OD of *P. fluorescens* grown in 4 mm sand and 3 mm glass beads pre-incubated with 50x DM (orange) or 0x DM (blue). *P. fluorescens* growth in different concentrations of DM without substrate are given as comparison. All ODs are means ± SEM, n = 3.

Table S1: Diffusion Parameters for Glass Bead Substrates of Varying Size[illegible]

Bead size (mm)	Diffusion rate (cm h^{-1})
3	21.6
2	4.48
1	1.56
0.5	1.02

Diffusion parameters calculated based on Congo Red diffusion through a column filled with glass beads of varying sizes.

Table S2: Metabolomics data

See excel file
