# Supplementary material for: Root morphology and exudate availability are shaped by particle size and chemistry in Brachypodium distachyon
Source: Plant Direct. 2020 Jul 2;4(7):e00207. doi: 10.1002/pld3.207 (PMC7330624; doi:10.1002/pld3.207)
Supplement: Supplementary file 3 — Supplementary Material 2 [file PLD3-4-e00207-s003.pdf]

Dear Dr. Lobet,

Thank you for the invitation to submit a reviewed version of our manuscript (Plant Direct: 2019-00319-T).
We are happy to see an overall positive response of both reviewers, and we are grateful for the comments on how to improve the structure of the manuscript.
We herewith submit a revised manuscript, with a restructured abstract, introduction and results section, as suggested by the reviewers. We reformatted the supplemental table as requested by a reviewer. As part of this process, we reprocessed all of the liquid chromatography data through an updated version of the software that automatically outputs these details. This required added a new author (SM Kosina). We also added another tab to the supplemental table with explanations.
We further incorporated the suggestions of both reviewers, and formulated detailed responses to their comments.
We feel that the manuscript is much improved now, and we are looking forward to your response.

Kind regards,

Joelle Sasse & Trent Northen

--- Reviewer comments:
Reviewer #1:

The authors performed a series of well-controlled experiments to determine how particle size and chemistry of the growth substrate shape root morphology, how exudate profile changes with particle size, and how root morphology and exudation are influenced by substrate chemistry. Furthermore, they assessed the effect of clay-sorbed metabolites on microbe growth. In the experiments performed, the authors were cautious to avoid confounding effect and therefore the data manage to answer some open questions regarding root exudation in different substrates with different particle sizes.

Dear reviewer 1,

Thank you for your positive feedback on our manuscript. We restructured the abstract, and the other sections of the article, as suggested. We feel that with your comments, the manuscript improved significantly. Please find detailed responses to your comments below (the line numbers refer to the tracked manuscript version).

- In my opinion, it would help to attract attention to this article, if the abstract was better structured; at the moment it's kind of convoluted, e.g. it's mentioned twice that root morphology is affected by substrate size, but exudation is not, and the open questions to be answered in this manuscript and why this is important remain elusive here, or rather are explained later on in the text.

Thank you for your feedback. We restructured the abstract as suggested:

Line 52: "Root morphology and exudation define a plants sphere of influence in soils. In turn, soil characteristics influence plant growth, morphology, root microbiome and

rhizosphere chemistry. Collectively, all these parameters have significant implications on the major biogeochemical cycles, crop yield and ecosystem health. However, how plants are shaped by the physiochemistry of soil particles is still not well understood. We explored how particle size and chemistry of growth substrates affect root morphology and exudation of a model grass. We grew *Brachypodium distachyon* in glass beads with various sizes (0.5, 1, 2, 3 mm), as well as in sand (0.005, 0.25, 4 mm) and in clay (4 mm) particles and in particle-free hydroponic medium. Plant morphology, root and shoot weight were measured. We found that particle size significantly influenced root fresh weight and root length, whereas root number and shoot weight remained constant.
Next, plant exudation profiles were analyzed with mass spectrometry imaging and liquid chromatography-mass spectrometry. Mass spectrometry imaging suggested that both, root length and number shape root exudation. Exudate profiles were comparable for plants growing in glass beads or sand with various particles sizes, but distinct for plants growing in clay. Clay particles were found to sorb 20% of compounds exuded by clay-grown plants, and 70% of compounds from a defined exudate medium. The sorbed compounds belonged to a range of chemical classes, among them nucleosides, organic acids, sugars, and amino acids. Some of the sorbed compounds could be de-sorbed by a rhizobacterium (Pseudomonas fluorescens WCS415), supporting its growth. This study demonstrates the effect of different characteristics of particles on root morphology, plant exudation and availability of nutrients to microorganisms. These findings further support the critical importance of the physiochemical properties of soils when investigating plant morphology, plant chemistry, and plant-microbe interactions."

- Line 92: it's not clear under which conditions a reduction in sugar exudation and an increase in amino acid exudation was observed

These were the same conditions as mentioned in the previous sentence. We restructured these sections of the introduction:

Line 103: "In contrast to particle chemistry, the effect of particle size on exudation is less clear. Maize growing in 1 mm glass beads exhibited a small reduction in sugar exudation and an increase in amino acid exudation compared to hydroponic controls (Boeuf-Tremblay et al., 1995), whereas another study featuring the same growth conditions found no change in carbon exudation (Groleau-Renaud et al., 1998)."

- Line 244: compared to, rather than compared versus

Thank you, the section was changed accordingly.

- Line 368: last word 'it' does not fit to sentence

Thank you, "it" was removed from the sentence.

Reviewer #2:

Review of Root morphology and exudate availability is shaped by particle size and chemistry in Brachypodium distachyon-Journal of Plant Direct

The authors invested the effect of growth medium with different particle sizes on some morphological features (such as root length, root biomass, shoot biomass) and exudation of a model grass Brachypodium distachyon. The objective of this study is interesting, and I

was highly motivated to read through their manuscript. The experimental setups and the findings are great and definitely worth to be published but this is a pity; I cannot refrain from emphasizing that the presentation of the manuscript needs careful revisions.
The introduction is poorly written and needs a serious revision. The authors gave scattered information throughout the introduction and did not provide a good link between how and why growth medium with different particle size may affect root exudation and root morphology. It also misses the acknowledgement of existing literature. I suggest the authors include the following topics: Give some hints in the introduction of why exudation (rate or quality) should be affected by different particle size? Is it related to the diffusion of sugars at the root-soil interface? Is it related to mechanical resistance? Is it related to microbial activity? Or it is related to the root growth rate? All these possibilities have been discussed and documented in the literature. I recommend the authors to read the following articles: DOI: 10.1007/s11104-017-3522-4, DOI: https://doi.org/10.1046/j.1365-3040.1999.00512.x; DOI: https://doi.org/10.1007/BF00009191); DOI: DOI: 10.1007/s11104-017-3424-5; DOI: https://doi.org/10.3389/fmicb.2014.00002.

The results section also needs careful revision. The results section everywhere throughout the text starts with some details related to the experimental or measurement procedure. I suggest that the authors move this information to the material and method where they belong. Here I gave you some examples that need to be revised lines 132:146; Lines 159:161; lines 177:180; Lines 220:228 and etc. The information given here is important I suggest that move them into the material and method.

The materials and methods section also needs revision. In addition to the comments above, I suggest that the authors provide more detail on the experimental method and the objectives of each measurement. I believe that a more comprehensive description of the measurement is particularly needed in their work because so many valuable measurements and it is a pity that the logics behind each measurement may not become clear to the readers. I gave you also some comment in the following to be included in this section.

Dear reviewer 2,

Thank you for your positive feedback on the manuscript, and the many thoughtful suggestions and comments to improve the article. We restructured the introduction as suggested, and incorporated the suggested literature and link between particle size and exudation. Further, we moved sections from results to materials & methods, as suggested, and the logic of the various experiments was explained in more detail in the materials& methods section. We also addressed your specific comments, see below for details (the line numbers refer to the tracked manuscript version).
We feel that with your comments, the manuscript improved significantly.

Specific comments:
Lines 83: why the authors use the term sandstones and not soil particle?

The authors of this publication referred to sandstones. We removed this part from the introduction.

Lines 82-84: I am not sure if this statement is in the context of their work? Why should one care of sandstone and rocks? I suggest replacing it with a more relevant statement about soil.

Thank you, we removed this section.

Lines 80-88: It is not clear what do the authors want to deliver here? The authors stated rather scattered information here and did not bring it to any context related to the root morphology and root exudation?

Thank you for this comment. We restructured the respective information in the introduction into two paragraphs, the first one focusing on how root morphology is shaped by soils, and the second on how exudation is shaped by soils:

Line 89: "Plant morphology and exudations can be influenced by both physical and chemical properties of soil particles. Typically, particles range from small (< 50 µm) to large (> 2 mm), and determine physical parameters such as water holding capacity of soils (Six et al., 2004; Six and Paustian, 2014; Rellán-Álvarez et al., 2016). It has been shown that 1 mm beads reduce root and shoot growth, elevate root:shoot ratios and alter root morphology of maize when compared to hydroponic growth (Veen, 1982; Boeuf-Tremblay et al., 1995; Groleau-Renaud et al., 1998). Root morphology can be altered by adsorption of root exudate metabolites to substrate such as activated carbon (Caffaro et al., 2011). Natural environments, such as soils can also affect both root exudate profiles and morphology (Neumann et al., 2014), however determination of causal factors may be confounded by variables such as granule size, chemistry, and microbial community composition. Soil minerals differ in structure (e.g. accessible surface areas) and surface charge, thereby governing their interaction with dissolved organic compounds (Swenson et al., 2015a). Results suggest that substrate chemistry can alter exudation. For example, aluminum ions present in stonewool are thought to increase exudation of organic acids in maize (Kamilova et al., 2006)."

Line 96-106: In this paragraph, I was expecting to see more information about the effect of growth medium on root exudate but surprisingly I saw a mixture of the effects on root morphology and root exudation. I suggest that the authors close the story of the effect of growth medium on root morphology in the previous paragraph and assigned this paragraph on the reported effect of growth medium on root exudation (from physical to chemical and biological effects).

We wanted to discuss how substrate chemistry can alter root morphology and exudation. We restructured these paragraphs to have a plant-centered focus, discussing first the effects of soil physiochemical properties on morphology, and then on exudation (Line 89, see above).

Lines 137: Here and elsewhere in the text, the authors used the term sand for substrates with the particle size of 5 µm. The particle with such a diameter are silt.

Thank you for pointing this out. We prefer to keep the term 'sand' also for the small particles, to indicate the similarity to the other sand substrates.

Line 148-149: Consider rephrasing " whereas the recovery rate from the 250 µm sand was approximately 30% lower, and from clay approximately 70% lower".

Thank you, we rephrased:

Line 140: “whereas the recovery rate from the 250 µm sand and from clay were lower by approximately 30% and 70%, respectively.”

Line 163: What does it mean less linear?

Thank you for pointing this out. We removed this phrase from the text.

Line 366-368: Why did the authors normalized the exudation per fresh root biomass and not the root length? I suggest normalizing it per root area or root length since they showed that different growth medium altered root morphology such as root diameter. If not justify your decision in the text.

Thank you for these comments. We now realize that it was not clear that we experimentally normalized the samples. This is a common best practice in metabolomics experiments because one cannot computationally normalize the values of metabolites that are not detected. Hence, we resuspended metabolite extracts in solvent volumes proportional to the root biomass prior to metabolomics analysis. The downside of this is that we are unable to computationally compare different normalization approaches and think that this would make for an interesting avenue for future research.
In our dataset, root weight, root length, and root numbers correlate (Figure S4). Thus, to speculate, it is likely that even with a different mode of normalization, similar results would have been obtained.

The text has now been revised to make this point clear:

Line 522: " Finally, samples were resuspended in 100% LC/MS grade methanol with 15 µM internal standards (767964, Sigma-Aldrich), with the solvent volume being proportional to the root biomass (0.77 g ml-1){Sasse:2018fq}. "

Line 371-376: the authors may refer to a recent study I came across DOI: 10.1007/s11104-017-3522-4. In this study, they showed that exudation sites were mainly at the root tip.

Thank you, we incorporated the reference:

Line 350: “In the literature, root tips are often mentioned as predominant sites of exudation for several reasons: i) cell wall suberization of this young tissue is still low (Jones et al., 2009), ii) exudates were imaged around root tips (Peters and Long, 1988)(Holz 2018) (…)”

Line 403:416: Is still the conclusion true if the authors normalized exudation per root length? Clarify it?

In metabolomics experiments, it is common to normalize samples prior to measurement. It is not possible to computationally normalize after measurement. Thus, we are unable to give a definite answer to this question.
However, as root weight, total root length and root numbers correlate in our experimental setup (Figure S4), we would not expect a completely different outcome with a different normalization method.

Line 530: Provide more details about this experiment. Under what condition this measurement was done? The medium was packed, or suspension was used? What was the ratio of substrate to different chemicals (mass of substrate versus the concentration of chemicals)? What was the concentration of the solution used here?

Thank you for asking for clarifications. The various substrates were packed as for the plant experiment under sterile conditions. The concentration of the chemicals was 20 µM, and the substrates were fully covered with the solution. We adjusted the text as follows:

Line 507: "The various substrates were sterilized, and the defined medium was prepared as a sterile, 20 µM equimolar solution. The substrates were fully submerged in the defined medium in sterile conditions, and incubated at 24°C for 8 hours. (…)"

Lines 519-522: Did the authors used a substrate with a fixed and uniform particle size? clarify it in the text.

The substrates were used as provided by the manufacturer. The glass beads were uniform in size as judged by eye (the manufacturer did not provide a size range or error margin for the beads). The natural substrates (clay, sand) varied more in diameter. Given are the manufacturers sizes, which are based on average particle diameter.

Line 535: What do the authors with recovery here? Is it a kind of desorption isotherm test?

In this experiment, defined medium was added to various substrates. The defined medium was pipetted off again after an incubation period. For substrates with large particle sizes, almost the complete volume of medium could be retrieved. For substrates with small particle sizes however, it was not possible to regain the complete volume (for example, 5 ml of medium was added, and it was possible to remove 3 ml by pipetting). We indicate this in the text:

Line 510: "The recovered volume was recorded, for substrates with smaller particle sizes, not the entire volume could be reclaimed."

Line 552: What do the authors mean here with substrate? Growth mediums with size?

We used a subset of substrates that were used for the plant growth assay. We now specify this in the text:

Line 528: "In the various experimental systems used here, exudation rates could be limited by diffusion. To determine the diffusion rates of various substrates, sterilized substrates (3 mm, 2 mm, 1 mm, 0.5 mm glass beads, 4 mm sand, 4 mm clay, or no substrate) were added to pipettes with a 50 ml volume. (…)"

Line 550: Clarify in more details the experimental setup used to measure the diffusion coefficient. Was the growth medium saturated? How long did it take? Do you neglect the microbial degradation (was it sterilized?), justify it? If not, the authors could not compare the results of different mediums since they will be at different water contents? Can you justify that the front did not move initially in space due to the addition of dye (mass flow)?

Thank you for asking for clarifications. The substrates were indeed saturated, and the timing of the experiment was in the same range as the exudate collection (exudates: 2 h, diffusion: 0-4.5 h). The experimental material was sterilized at the beginning, but the experiment was not conducted in a sterile environment. Due to the timing of the experiment, we assume that microbial growth during the experiment can be neglected. It is likely that in the initial phase of the experiment, mass flow added to the dye movement.

We adjusted the text as follows:

Line 528: “In the various experimental systems used here, exudation rates could be limited by diffusion. To determine the diffusion rates of various substrates, sterilized substrates (3 mm, 2 mm, 1 mm, 0.5 mm glass beads, 4 mm sand, 4 mm clay, or no substrate) were added to pipettes with a 50 ml volume. The pipettes were sealed at the bottom with parafilm, placed vertically, 50 ml of substrate was added, and approximately 25 ml of sterilized 0.5x MS was added to fully immerse submerse the substrate (there was no correlation between the amount of 0.5x MS added and the volume needed to immerse the substrate). The experimental setup was sterile, but the experiment was conducted in non-sterile conditions. Congo red 4B (C6277, Sigma-Aldrich) was solubilized in water at a concentration of 20 mg ml-1, and 250 µl of the dye was added simultaneously to pipettes containing the various substrates. The front of the dye was followed recorded over time up to 4.5 h. Initially, the movement of the dye front was supported by mass flow (gravity).”

Line 562: how do the author justify the use of this exponential equation. I would rather suggest them to solve a diffusion equation and get the diffusion coefficient or stay on with the calculation of D_eff from Equation in line 565. This approximation would be correct if their system could hold the assumption of the steady-state condition? Does it? How about microbial degradation? I am not familiar with the concept of diffusion rate with unite of cm/h. Do the authors mean diffusional permeability constant? Please indicate the measured data in Fig. 1a with dots and show also your fitting with lines.

Thank you for your observation. Indeed, there are multiple ways to calculate diffusion coefficients. As the diffusion calculations did not add much to the manuscript, we decided to delete this part.
Further, we now indicate the measured data in Fig 1a with dots, in addition to the lines.

Table S2, what does this table show? add some explanation to this table. The headings Retention time Median RT shi m/z to which column are they referring? Unite of Retention time? Median of what?

Thank you for pointing this is out. We have reformatted the table and added comments to clarify the content. We also included additional evidence supporting identifications.

Fig1 b: add the legend. Are these lines the measurements or fitting? Show the measurement points with dots.

The lines are based on measurements. The single points are now indicated with dots, thank you.

Fig. 3: It is not clear what do these images show? What are the images in the first row and what are the ones in the second row? What are the numbers given here? I see that more detail is given in Fig. S5.

We added the method (Mass Spectrometry Imaging) for additional explanation:

Line 714: “Root-associated ion patterns (50-2000 Da) of several ions were observed with Mass Spectrometry Imaging. The patterns of several ions are displayed here, with their weight indicated above the panels. (…)”

Figure S3: Use a different colouring pattern here. It is not possible to distinguish the three different root orders here.

We agree that the colors used are not the best choice. Unfortunately, the colors are determined by the software used (Smartroot, https://smartroot.github.io/), and we are not able to change them. We hope that with appropriate monitor color settings, it will be possible to distinguish the green from yellow.
